# Deficiency of the palmitoyl acyltransferase ZDHHC7 modulates depression-like behaviour in female mice after a mild chronic stress paradigm

**DOI:** 10.1038/s41398-025-03240-7

**Published:** 2025-01-24

**Authors:** Christa Hohoff, Nicole Kerkenberg, Mingyue Zhang, Weronika Palkowska, Lydia Wachsmuth, Maja Peng, Lena Stiehl, Christiane Schettler, Johannes C. S. Zang, Andreas Huge, Evgeni Ponimaskin, Cornelius Faber, Bernhard T. Baune, Weiqi Zhang

**Affiliations:** 1 Department of Psychiatry, University of Münster, 48149 Münster, Germany; 2 Otto Creutzfeldt Center for Cognitive and Behavioral Neuroscience, University of Münster, 48149 Münster, Germany; 3 Clinic of Radiology, University of Münster, 48149 Münster, Germany; 4 Department of General Internal Medicine and Psychosomatics, University Hospital Heidelberg, Heidelberg, Germany; 5 Core Facility Genomics, University of Münster, 48149 Münster, Germany; 6 Cellular Neurophysiology, Hannover Medical School, 30625 Hannover, Germany; 7 Department of Psychiatry, Melbourne Medical School, University of Melbourne, Parkville, VIC 3010 Australia; 8 Florey Institute for Neuroscience and Mental Health, University of Melbourne, Parkville, VIC 3010 Australia

**Keywords:** Molecular neuroscience, Depression

## Abstract

Chronic stress (CS) is a debilitating condition that negatively affects body and brain. In mice, CS effects range from changes in behaviour and brain microstructure down to the level of gene expression. These effects are partly mediated by sex and sex steroid hormones, which in turn are affected by the palmitoyl acyltransferase ZDHHC7. ZDHHC7 might modulate also the response to CS via palmitoylation of sex steroid hormone receptors and other proteins critical for neuronal structure and functions. Therefore, we aimed to investigate the role of ZDHHC7 in response to CS on different system levels in a mouse model of *Zdhhc7*-deficiency. Female and male *Zdhhc7*-knockout (KO) and -wildtype (WT) mice underwent a four-week-mild CS paradigm or non-stress control (C) condition. After C or CS, behaviours, hippocampal microstructures (via MRI-based diffusion tensor imaging) and brain gene expression profiles (via mRNA-seq transcriptomics) were investigated. Analyses focused on effects of genotype (KO vs. WT) or condition (C vs. CS) separately in both sexes. Our results revealed significant effects particularly in females. Female KOs displayed increased locomotion and reduced depression-like behaviour after CS (KO vs. WT, C vs. CS: *p*_*all*_ < 0.05). Hippocampal fibres were reduced in female KOs after C (KO vs. WT: *p*_*all*_ < 0.05) but in female WTs after CS (C vs. CS: *p*_*all*_ < 0.05). Furthermore, female KOs showed increased *cortistatin* expression after CS (C vs. CS: mRNAseq and qPCR *p*_*all*_ < 0.05). In sum, *Zdhhc7*-deficiency reduced depression-like behaviours, prevented hippocampal fibre reduction and upregulated *cortistatin* after CS. It seemed to be related to a sex-specific stress response and may reveal genetic factors of CS-resilience in female mice.

## Introduction

Chronic stress describes a state of persistent stress reactions occurring primarily when negative stressors act long-term on individuals. Accompanying persistently increased glucocorticoid levels have negative effects on body and brain [1], which can result in e.g., mitochondrial dysfunction, cell death, neuronal damage and general brain pathologies [2, 3]. In stress vulnerable individuals, chronic stress may result in the development of psychiatric and neurodegenerative disorders such as anxiety disorders, depression, Alzheimer’s and Parkinson disease [3–5]. Similarly, chronic stress triggers anxiety- and depressive-like behaviour in rodents and impairs cognition-like behaviour such as spatial response [6–9]. Stress induced behavioural changes in humans and animals are linked particularly to the prefrontal cortex (PFC) and the hippocampus as parts of the limbic system (e.g., [4, 10–15]). The PFC regulates executive cognition-related processes and modulation of anxiety- and depression-like behaviours via numerous connections to other cortical and subcortical brain areas (e.g., [7, 16–19]). The hippocampus mediates cognitive, mainly spatial, memory functions together with emotional, mainly stress-related, responses due to its high concentration of corticosteroid receptors as well as functional and structural connectivity with the PFC [15, 20–25]. In both, PFC and hippocampus, chronic stress leads to microstructural and reorganisational changes due to e.g. decreased neurogenesis, reduced dendrites and altered synaptic terminal structure [23, 26–30]. Further, stress triggers gene expression and an upregulation of genes involved in neuronal structure, function and synaptic plasticity in PFC and hippocampus [31, 32]. Such stress induced changes on behavioural, brain microstructural and gene expression levels are, among others, also affected by sex and sex steroid hormones [33–39].

The palmitoyl acyltransferase ZDHHC7 is widely expressed in brain (e.g. cortex, hippocampus) and periphery (e.g. liver, kidney, testis) [40]. It palmitoylates sex steroid hormone receptors involved in emotion, cognition, neurogenesis and myelination [41–44], together with palmitoylation of other synaptic and extrasynaptic proteins critical for neuronal structure and function [45–47]. During palmitoylation, ZDHHC7 enzymes add 16-carbon palmitic acid to specific cysteine residues of target proteins in a reversible way [46], which increases hydrophobicity and facilitates membrane localisation of target proteins, allowing an additional non-genomic and rapid signal response at the cell membrane [42, 43, 46]. In case of sex steroid hormone receptors, this rapid signalling modulates also gene expression and early hippocampus organisation in a sex-specific way [43, 48–50]. Furthermore, our recent data from a mouse model with deficiency in the murine *Zdhhc7* gene revealed sex-specific behavioural, brain microstructural and functional changes in a control (unstressed) situation [51], as well as after acute stress [52]. This raises the question, whether and how *Zdhhc7*-deficiency would also affect response to chronic stress in male and female mice. However, to the best of our knowledge, so far no study analysed the impact of *Zdhhc7*-deficiency on behaviour, brain microstructure and transcriptome-based gene expression after chronic stress.

Therefore, the present study aimed to investigate *Zdhhc7*-deficiency-mediated male and female mouse phenotypes after mild chronic stress on different system levels, including locomotion-, emotion- and basal cognition-related behavior, brain microstructure, as well as transcriptomic and subsequent candidate gene expression profiles. Based on the above-mentioned previous findings we hypothesized that *Zdhhc7*-deficiency alters the response of mice to mild chronic stress on a systems level and in a sex-specific manner.

## Materials and methods

### Experimental animals

Altogether 182 mice of our *Zdhhc7*-deficiency model [51] were used (47 female WTs, 47 female KOs, 44 male WTs, 44 male KOs). They were bred in-house (central animal facility (ZTE) of the University of Münster (Germany)) based on heterozygous *Zdhhc7* mouse crosses. All mice were weaned at 4-5 weeks of age and kept in same-sex littermate groups in individually ventilated cages (IVC) under controlled ZTE conditions until start of the experiments (see below). The present work was in accordance with all current regulations covering animal experimentation in Germany and the EU (European Communities Council Directive 2010/63/EU). All study-specific experiments and procedures were approved by the local responsible government authority (LANUV-NRW, Germany; Az84-02.04.2016.A416) and all efforts were made to minimize animal suffering.

### Experimental design

At 8 weeks of age *Zdhhc7*-WT and -KO mouse littermates were transferred to an experimental room where they remained until the end of the trial at 15–16 weeks of age. During this experimental phase mice were housed in Makrolon Type II L cages (37 x 21 x 14 cm) at controlled conditions (1 same-sex WT-KO pair (2 mice) per cage; filtertops without IVC; temperature 22 ± 2 °C; humidity 55 ± 10%; 12:12 light-dark cycle with lights on at 6 am). Sawdust was given as bedding material and access to tab water and food (standard diet 1324; Altromin, Lage, Germany) *ad libitum*. One paper house (smart homes; Datesand Group, Manchester, UK) and 6 pressed cotton pads (Zelletten, 5x4cm; Lohmann & Rauscher, Rengsdorf, Germany) were provided as nesting material once per week after transferring mice into fresh cages. All mice were handled daily (score sheet health checks) and weighed twice a week by experienced experimental personnel. After two weeks of habituation at 10 weeks of age, WT-KO littermate pairs were alternately assigned (based on numerical mouse IDs given at birth by ZTE in ascending order) to the non-stress control (C) group (female WT: *n* = 24; female KO: *n* = 24; male WT: *n* = 22; male KO: *n* = 22) or to the chronic stress (CS) group (female WT: *n* = 23; female KO: *n* = 23; male WT: *n* = 22; male KO: *n* = 22) with males kept in single housing thenceforth. CS mice were exposed to a 4 week stress paradigm including unpredictably applied day-time stressors (3 h restraint stress; 3–5 h rat odour; 3–9 h tilted (30°) home cage; 3–9 h home cage without nesting material), night-time stressors (15–16 h humid bedding or tilted home cage or home cage without nesting material), and weekend stressors (62–65 h reversed light-/dark cycle). Only the rat odour stressor was applied in a separate laboratory room, all other stressors in the experimental housing room in which the control group meanwhile was kept under control conditions in their home cages. At 14 weeks of age all mice were behaviourally tested (see below, once per mouse and test) and afterwards alternately assigned (based on numerical mouse IDs, see above) to different final experimental sets. For these final sets, mice were sacrificed at 15–16 weeks of age to collect biological materials for either brain microstructure analyses or gene expression analyses (both see below) or further analyses which are not part of the present study. All experimental sets were performed only once without replication as approved by the local responsible government authority (LANUV-NRW, Germany; Az84-02.04.2016.A416) to minimize animal numbers. Sample size estimation was based on our previous studies [51, 52].

### Behavioural testing

All mice (*n* = 182) went through a series of behavioural tests, which were conducted in the above mentioned experimental room on three successive days. The tests were performed as previously described [52] in the following order (for an illustration of the timeline of behavioural testing see Suppl. Fig. 1): On the first day in the morning (at approx. 8 a.m.) the elevated plus maze (EPM) test for the evaluation of anxiety-like behaviour and locomotion as well as the spontaneous alternation test (SAT) for the evaluation of basal spatial working memory and locomotion. On the first day in the afternoon (at approx. 2 p.m.) the social interaction (SI) test for the assessment of depression-like behaviour was operated. On day two in the morning (at approx. 10 a.m.) the nest building (NB) test for assessment of attention and general stress load started and lasted until day three in the morning (scoring after 1 h, 3 h, 7 h and 24 h). On day three in the afternoon (at approx. 3 p.m.) the tail suspension (TS) test was conducted again for the evaluation of depression-like behaviour. Behaviours were recorded automatically (tracking software ANY-maze v. 6.33; Stoelting Europe, Dublin, Ireland) in all tests, but technical failures in EPM (1 female WT-KO pair and 1 male WT-KO pair, both in C), SAT (1 male WT-KO pair in C) and SI (1 male WT-KO pair in CS) led to exclusion of these defective recordings. This resulted in analysable recordings for 21 to 24 biological replicates per group, in EPM for 178 mice (C: 23 female WTs, 23 female KOs, 21 male WTs, 21 male KOs; CS: 23 female WTs, 23 female KOs, 22 male WTs, 22 male KOs), in SAT for 180 mice (C: 24 female WTs, 24 female KOs, 21 male WTs, 21 male KOs; CS: 23 female WTs, 23 female KOs, 22 male WTs, 22 male KOs), in SI for 180 mice (C: 24 female WTs, 24 female KOs, 22 male WTs, 22 male KOs; CS: 23 female WTs, 23 female KOs, 21 male WTs, 21 male KOs), as well as in NB and TS each for 182 mice (C: 24 female WTs, 24 female KOs, 22 male WTs, 22 male KOs; CS: 23 female WTs, 23 female KOs, 22 male WTs, 22 male KOs).

### Brain microstructure analysis

A subset of 40 mice (20 females, 20 males) were anesthetized intraperitoneally, transcardially perfused and examined using an MR protocol as detailed previously [51, 52]. Briefly, mouse brains enriched with contrast agent (2 mmol/L Magnevist, Bayer Pharma AG, Berlin, Germany) were scanned on a 9.4 T small animal imaging system with helium-cooled surface coil (Bruker Biospin, Ettlingen, Germany) for MRI-based diffusion tensor imaging (DTI). Deterministic [53] fibre tractography (DTI&Fiber Tool; [54]; https://www.uniklinik-freiburg.de/mr-en/research-groups/diffperf/fibertools.html) used the same medioventral hippocampal regions of interest in left and right hemisphere as aforementioned and applied [51, 52]. Due to technical failures this resulted in fibre numbers and lengths (mean) analysable for 34 mice (4 to 5 biological replicates per group; C: 5 female WTs, 5 female KOs, 4 male WTs, 4 male KOs; CS: 4 female WTs, 4 female KOs, 4 male WTs, 4 male KOs).

### Gene expression analyses

A subset of 52 mice (30 females, 22 males) were anaesthetized with 5% isoflurane (Cp-Pharma, Burgdorf, Germany) for 50 s, decapitated 10 s later and prepared to immediately dissect medioventral hippocampi and medial prefrontal cortices (mPFC). Six to 8 min. after decapitation, dissected tissues were stored in RNA*later* (Thermo Fisher Scientific, Darmstadt Germany; 24 h at 4 °C and subsequently at −80 °C). RNAs were extracted from medioventral hippocampi using Direct-zol RNA MicroPrep Kit (Zymo Research, Irvine, USA) and from mPFCs using Direct-zol RNA MiniPrep Kit (Zymo Research, Irvine, USA), both separately from each brain hemisphere per mouse. Extractions resulted in samples with sufficient yield (> 180 ng total RNA) and purity (260/280 > 1.9), both measured by NanoDrop One/OneC (Thermo Fisher Scientific), for medioventral hippocampus and mPFC in right hemispheres of 40 mice. RNA aliquots of these 40 mice (4 to 6 biological replicates per group; C: 6 female WTs, 6 female KOs, 4 male WTs, 4 male KOs; CS: 6 female WTs, 6 female KOs, 4 male WTs, 4 male KOs;) were further processed in the Core Facility Genomics (University of Münster, Germany). Quality measures (TapeStation RNA System or Bioanalyzer with Pico Chip; both Agilent, Santa Clara, USA) revealed mean RIN values of 7.6 (range 6.5–8.6) for medioventral hippocampus RNAs and 8.2 (range 7.4–8.6) for mPFC RNAs. For library preparation with the NEBNext Ultra II RNA directional kit (BioLabs, Frankfurt a. Main, Germany) mean input RNA amounts were 211 ng (range 110–250) for medioventral hippocampus and 486 ng (range 200 to 500) for mPFC. Sequencing reactions (25 Mio. single reads, 75 bp) were performed on a NextSeq500 System (Illumina, San Diego, USA), followed by demultiplexing and conversion of samples to fastq data (bcl2fastq2 conversion software v. 2.20; Illumina) and quality control (FastQC v. 0.11.9; Babraham Bioinformatics, Cambridge, UK). After adapter trimming and read filtering (Trimmomatic v. 0.38; [55]) the resulting reads were aligned (HISAT2 v. 2.1.0; [56]) to the reference genome (Ensembl GRCm38), sorted (SAMtools v. 1.9; [57]) and counted into genomic features (htseq-count v .0.11.1; [58]; GEO submission number is GSE281404) for later statistical analyses (see below).

Subsequently, top four candidates of differentially expressed genes (DEG) were selected based on statistically significance and literature-based relevance for the nervous system and were independently validated by quantitative real-time PCR (qPCR). For this, the remaining RNA amounts not used for library preparations were cDNA-synthesised (SuperScript IV VILO Master Mix with ezDNase Enzyme; Thermo Fisher Scientific) with input amounts of 2.1 ng for *n* = 40 medioventral hippocampus samples and 10.6 ng for *n* = 40 mPFC samples. The qPCRs were run in triplicate (setup on a Freedom EVO 150 system; Tecan, Crailsheim, Germany) using TaqMan Fast Advanced Master Mix (Thermo Fisher Scientific) and TaqMan Gene Expression Assays (DEGs: Mm00480633_m1, Mm01253178_m1, Mm00432631_m1, Mm00484202_m1; *Gapdh*: Mm99999915_g1; Thermo Fisher Scientific) on a 7900HT qPCR instrument (Applied Biosystems, Thermo Fisher Scientific) following the manufacturer’s instructions. Relative expression of DEGs was analysed with RQ Manager v. 1.2.1 (Thermo Fisher Scientific) and normalised to endogenous control gene *Gapdh* based on average delta Cts and fold change calculation (2^-ΔΔCt^).

### Statistical analyses

All data sets (outcome measures) from behavioural testing, brain microstructure analysis and qPCR-based gene expression analyses were statistically tested using SPSS (version 28, IBM, Ehningen, Germany). Respective data distribution was tested group-wise (C or CS in WT or KO female (f) or male (m) mice) in all eight groups (1. C-WT-m, 2. CS-WT-m, 3. C-KO-m, 4. CS-KO-m, 5. C-WT-f, 6. CS-WT-f, 7. C-KO-f, 8. CS-KO-f). We checked for possible outliers using Cook distances (cut-off value = 1 with respect to https://statistikguru.de/ (© 2015 – 2024 W.A. Hemmerich — StatistikGuru Version 1.96) and leverage values (cut-off value = 0.2 with respect to Huber 1981 as mentioned in StatistikGuru Version 1.96). We then tested outcome measures in all eight groups for normality (Shapiro-Wilk test) and for normality of residuals (Kolmogorov-Smirnov test with Lilliefors significance correction; StatistikGuru Version 1.96) followed by testing homoscedasticity in each of the three effector groups (condition, genotype, sex) using Levene test of homogeneity of variance (based on median; StatistikGuru Version 1.96). Due to detected violations of normality and homoscedasticity in all of the eight groups, pairwise non-parametric testing (Mann-Whitney-U-Test) of outcome measures was performed. The alpha-level of statistical significance (two-sided) was set to 0.05 for analyses of genotype (WT vs. KO) or condition (C vs. CS) separately in female and male *Zdhhc7* mice. Transcriptomic-based analysis of differential gene expression was performed with the R package DESeq2 (v. 1.24.0; [59]) that is based on a model using the negative binomial distribution. After Normalisation and log2 Fold Change calculation the Wald test was applied for statistical analysis with correction for multiple testing by the Benjamini-Hochberg method. Genes with a false discovery rate FDR (*p* adj.) < 0.05 were considered differentially expressed. All figures were produced in excel and represented group means in bars, error bars as standard error mean ( ± SEM) and individual data points as dots.

## Results

### *Zdhhc7*-KO females showed reduced depression-like behavior after CS exposure

Behaviour of *Zdhhc7* mice was assessed after C or CS condition using the tests SAT and EPM for evaluation of locomotion, SI, TS and EPM for emotion-like behaviour as well as SAT and NB for basal cognition and overall stress load.

**Fig. 1:**
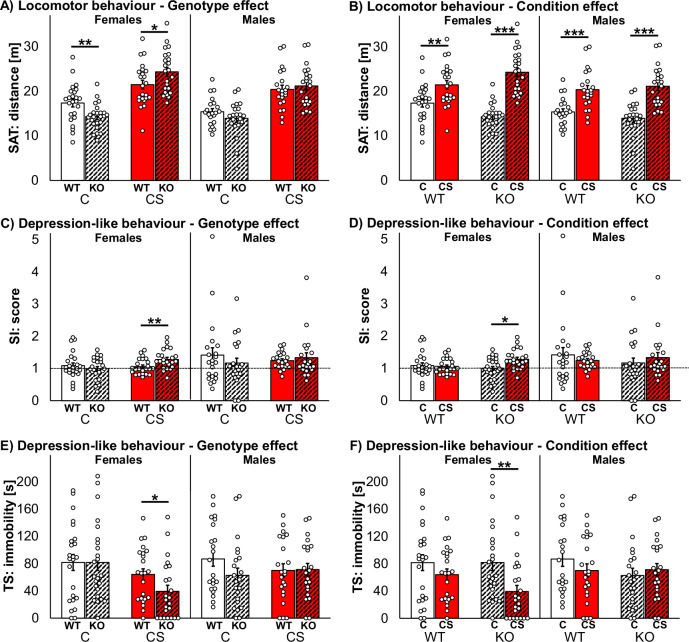
Effects of *Zdhhc7*-deficiency, chronic stress and sex on behaviour. *Zdhhc7* wildtype (WT) and knockout (KO) mice after control (C) or chronic stress (CS) conditions revealed differences in locomotor behavior with respect to genotype (**A**) or condition (**B**), and differences in depressive-like behaviour regarding genotype (**C**, **E**) or condition (**D**, **F**). Bars represent group means with standard error mean (±SEM) and individual data points as dots in spontaneous alternation test (SAT), social interaction test (SI) and tail suspension test (TS). Asterisks depict the level of significance (*p < 0.05; **p < 0.01; ***p < 0.001); sample sizes were *n* = 22–24 per group.

Locomotor behaviour was significantly influenced by genotype in female mice (Fig. 1A), that is, WTs traveled longer distances than KOs after C (SAT: *z* = −2.66, *p* = 0.008), but shorter distances than KOs after CS (SAT: *z* = 2.04, *p* = 0.041). Locomotion was further significantly influenced by condition in both sexes (Fig. 1B), i.e. females and males of both, WT and KO, traveled longer distances after CS than after C (SAT *p*_*all*_ < 0.01, EPM: *p*_*all*_ ≤ 0.001; for statistical details of all behavioural tests after C or CS condition see Suppl. Tab. 1).

Emotion-like behaviour was significantly influenced by genotype in female mice (Fig. 1C, E). After CS, interaction scores were higher in KOs vs. WTs (SI: *z* = 2.58, *p* = 0.010) and immobility times were shorter in KOs vs. WTs (TS: *z* = −2.24, *p* = 0.025), both indicating less depression-like behavior in KO females. Emotion was further significantly influenced by condition in female KOs (Fig. 1D, F). They scored higher in social interaction (SI: *z* = 2.55, *p* = 0.011) and spend less time immobile (TS: *z* = −2.83, *p* = 0.005) after CS vs. C, again indicating reduced depression-like behavior in KO females. In contrast, anxiety-like behaviour was only by trend influenced by condition in female WTs (less EPM open arm time after CS vs. C; Suppl. Tab. 1).

Basal cognition-like behaviour and overall stress load was significantly influenced only by condition in both sexes (Suppl. Tab. 1). At the beginning of the NB test phase, both female and male KO mice reached significantly lower scores after CS vs. C (males after 1 h: *z* = −2.04, *p* = 0.042; females after 3 h: *z* = −2.84, *p* = 0.005). Towards the ending of the NB test phase males performed significantly better after CS than after C (WTs after 7 h: *z* = 2.84, *p* = 0.004; KOs after 7 h: *z* = 2.47, *p* = 0.013, and after 24 h: *z* = 2.47, *p* = 0.013), indicating better attention and lower stress load.

### *Zdhhc7*-KO females revealed reduced hippocampal fibres but no fibre reduction after CS

Hippocampal microstructure of *Zdhhc7* mice was assessed after C or CS condition using left and right hemisphere medioventral hippocampus regions to evaluate fibre numbers and fibre lengths.

**Fig. 2:**
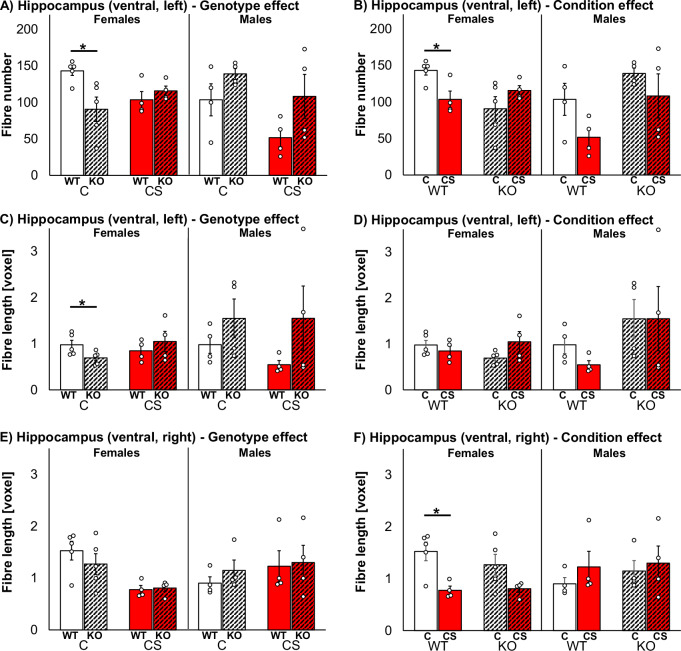
Effects of *Zdhhc7*-deficiency, chronic stress and sex on brain microstructure. *Zdhhc7* wildtype (WT) and knockout (KO) mice after control (C) or chronic stress (CS) conditions revealed differences in hippocampal fibres with respect to genotype effect (**A**, **C**, **E**) or condition effect (**B**, **D**, **F**). Bars represent group means with standard error mean (±SEM) and individual data points as dots in left or right ventral hippocampus regarding fibre numbers or lengths (named in respective plots). Asterisks depict the level of significance (*p < 0.05); sample sizes were *n* = 4–5 per group.

Fibre numbers were significantly influenced by genotype in left medioventral hippocampus of females (Fig. 2A). After C, female KOs revealed significantly less fibres compared to WTs (*z* = 2.20, *p* = 0.032). Fibre numbers were also significantly influenced by condition in left medioventral hippocampus in females (Fig. 2B), i.e. WTs showed lower fibre counts after CS vs. C (*z* = −2.21, *p* = 0.032; for statistical details of all medioventral hippocampus fibre tests after C or CS condition see Suppl. Tab. 2).

Fibre lengths were as well significantly influenced by genotype in left medioventral hippocampus in females (Fig. 2C). After C, KOs revealed shorter fibres compared to WTs (*z* = 2.19, *p* = 0.032), again indicating different brain microstructural organisation in KO vs. WT females. Fibre lengths were further significantly influenced by condition in right medioventral hippocampus in female WTs (Fig. 2F). They revealed shorter fibres after CS vs. C (*z* = −2.21, *p* = 0.032), indicating brain microstructural impairment in response to CS in female WTs.

### *Zdhhc7*-KO females revealed higher cortistatin gene expression after CS vs. C

Finally, transcriptomic mRNA sequencing profiles of *Zdhhc7* mice were assessed after C or CS condition to evaluate medioventral hippocampus and mPFC gene expression.

**Fig. 3:**
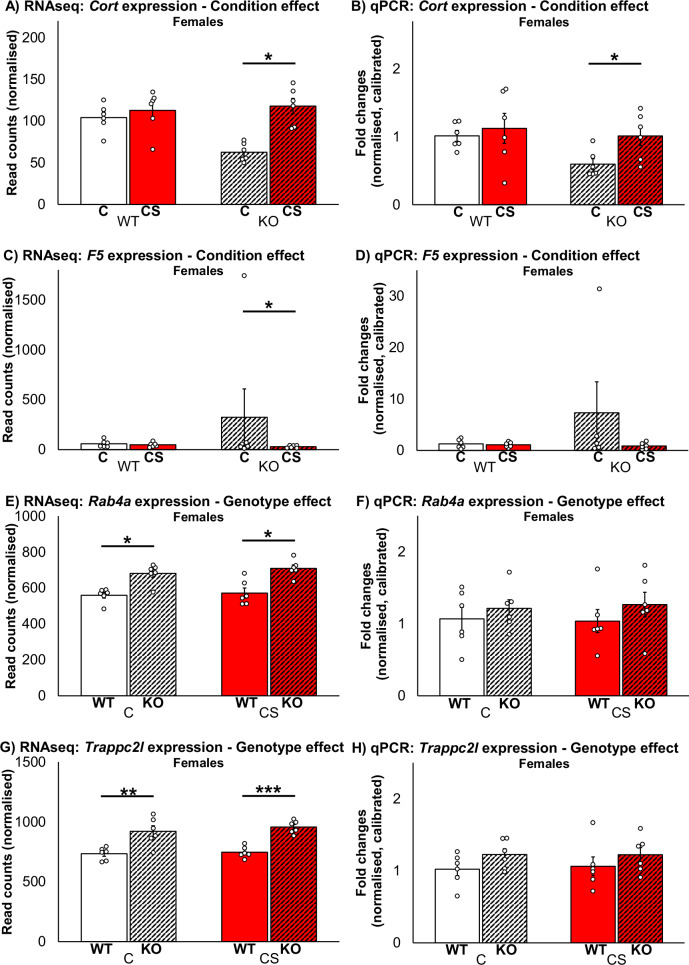
Effects of *Zdhhc7*-deficiency, chronic stress and sex on hippocampal gene expression. *Zdhhc7* wildtype (WT) and knockout (KO) mice after control (C) or chronic stress (CS) conditions revealed differences in hippocampal gene expression with respect to condition effect (**A**–**D**) or genotype effect (**E**–**H**). Bars represent group means with standard error mean (±SEM) and individual data points as dots regarding mRNA sequencing based expression (**A**, **C**, **E**, **G**) or regarding qPCR based relative expression calibrated to the control group “WT females after C” (**B**, **D**, **F**, **H**). Asterisks depict the level of significance (*p < 0.05; **p < 0.01; ***p < 0.001)); sample sizes were *n* = 5–6 per group.

In medioventral hippocampus, 20 genes survived adj. FDR correction after Wald statistical testing and were identified as differentially expressed (for genetically and statistical details of all DEGs see Suppl. Tab. 3 as well as Suppl. Figs. 2, 4 and 5). Of these DEGs, 16 were significantly influenced by genotype. *Zdhhc7* occurred as top DEG downregulated in KOs vs. WTs in both, conditions and sexes (*p*_all_(adj) < 0.001), thus confirming the knockout in our mouse model. Further DEGs significantly downregulated in KOs vs. WTs were *Gm42047* (both, conditions and sexes: *p*_all_(adj) ≤ 0.003) and *Aprt* (females after C: *p*(adj) < 0.05). Significant upregulation in KOs vs. WTs occurred in the DEG *Trappc2l* in females after C and after CS (*p*_both_(adj) ≤ 0.002; Fig. 3G) and in males after CS (*p*(adj) < 0.05). Further DEGs significantly upregulated in KOs vs. WTs were *Rab4a* and *Col8a1* in females after C as well as *Rab4a* and *Spg7* in females after CS (*p*_all_(adj) < 0.05; for *Rab4a* see Fig. 3E). Among genotype, gene expression in medioventral hippocampus was significantly influenced by condition in 4 genes and only in females. The DEGs *F5* and *Rps13-ps1* were significantly downregulated in KOs after CS vs. C (*p*_both_(adj) < 0.05; for *F5* see Fig. 3C), whereas *Cort* was significantly upregulated in this group comparison (*p*(adj) < 0.05; Fig. 3A). The DEG *Rps13-ps1* was further downregulated in WTs after CS vs. C (*p*(adj) < 0.05).

In mPFC, 13 genes were identified as DEGs (for genetically and statistical details see Suppl. Table 3 as well as Suppl. Figs. 3, 4 and 6), of which 10 were significantly influenced by genotype. Again, *Zdhhc7* occurred as top DEG downregulated in KOs vs. WTs in both, conditions and sexes (*p*_all_(adj) < 0.001), followed by *Gm42047* (both conditions and sexes: *p*_all_(adj) ≤ 0.001). Additional DEGs were identified in male KOs vs. WTs after C condition as downregulated (*Cyp2g1*: *p*(adj) < 0.001) or upregulated (*Rab4a*: *p*(adj) ≤ 0.003). Gene expression was further influenced by condition. The DEG *Rps13-ps1* was downregulated in both, WT and KO females after CS vs. C condition (*p*_both_(adj) ≤ 0.01), whereas *Cyp2g1* was identified as upregulated in KO males after CS vs. C (*p*(adj) < 0.001).

Together, particularly the DEGs *Trappc2l*, *Rab4a*, *F5* and *Cort* appeared promising and have therefore been selected for validation of transcriptomic results by real-time qPCR experiments. The qPCR analyses confirmed the DEG *Cort* as significantly upregulated in female KOs after CS compared to C in medioventral hippocampus (*z* = −2.08, *p* = 0.041; Fig. 3B), while the other three genes remained unvalidated (Fig. 3D, F, H).

## Discussion

The present study investigated whether and how the palmitoyl acyltransferase ZDHHC7 modulates the response to CS on different system levels in a mouse model of *Zdhhc7*-deficiency. We detected differences between genotypes (WT vs. KO) and/or condition (C vs. CS) particularly in female mice, e.g. with respect to locomotion, hippocampal fibres, depression-like behaviours, and *Cort* gene expression. In contrast, male mice revealed differences with respect to locomotion and basal cognition-like behaviour only after C vs. CS. *Zdhhc7*-deficiency might thus be related to a sex-specific stress response with greater modulation in females.

### The impact of *Zdhhc7* genotype on female mice

Females were influenced by genotype during C exposure with respect to locomotor behaviour and brain hippocampal fibres. Locomotion was increased in WTs (vs. KOs) after C, which is opposite compared to previous studies [51, 52]. Previously detected increased locomotion of *Zdhhc7* KO females was assumed as behavioural disinhibition and increased exploration because of the simultaneously observed reduced anxiety [51]. In our present study, this was not the case, possibly due to alteration in experimental settings. Our present behavioural testing involved mice at an age of 15 weeks (4 months) compared to 10 weeks (2.5 months) in Hohoff et al. [51] and 9 weeks (2 months) in Kerkenberg et al. [52]. Age-related behavioural changes are described for mice, involving decreased locomotor activity to novel environments in 4–5 months old mice vs. 2–3 months old ones [60]. In addition, we detected more and longer hippocampal fibres in female WTs vs. KOs after C, in line with previous observations from Kerkenberg et al. [61]. Therein, the authors detected increased brain fibres at 14 and 17 weeks of age in *Zdhhc7* WTs (vs. KOs). They suggested normally ongoing brain microstructural changes in early adulthood in WTs, in contrast to impaired fibre development in KOs at 14 and 17 weeks of age [61]. Such impaired fibre development in KOs might be reflected by the reduced fibres in our 15 week old KOs (vs. WTs), which might be responsible also for the reduced locomotion in our KOs (vs. WTs) after C. In line with this observation, Lu and colleagues [62] reported reduced exploration and locomotion (among other findings) in aged mice and suggested altered synaptic signaling as the cause for this. Since ZDHHC7 palmitoylates target proteins critical for e.g. neurogenesis, myelination, neuronal structure and function (c.f. introduction), decreased palmitoylation of critical proteins should then enhance the age effect compared to WTs and led to our observed differences.

### The impact of condition (C or CS) on female mice

Females were further influenced by condition with respect to locomotion and hippocampal fibres in the present study. Both, WT and KO females showed higher levels of locomotion after CS than after C, similar to other studies, which reported increased locomotion after acute or chronic stress paradigms [8, 63]. Both studies point to a close connection of locomotion and anxiety, for which our present study provided at best weak indications in female WTs. They tended to increased anxiety-like behavior in the EPM after CS and might thus have displayed a mild form of anxiety-based escape behavior in response to CS as described by Brinks et al. [63]. In addition, hippocampal brain fibres were reduced in female WTs after CS vs. C. Such CS induced microstructural changes are in line with other studies, which reported high sensitivity of the hippocampus to CS and as a result reduced neurogenesis, dendrites, pathways, connectivities and synaptic functions (reviewed in e.g., [64, 65]. Together, behavioural and brain microstructural changes of our female WTs might indicate a mild CS-susceptible response. This, however, was shown in a less distinct way than previously assumed (c.f. introduction) and reported (e.g., [6–9, 66], maybe due to the shorter duration of our CS paradigm (4 weeks vs. 6 weeks in other studies) in combination with milder stressors (vs. e.g. chronic social defeat or restrain stress; c.f., [7, 8, 66].

### The impact of *Zdhhc7* genotype and condition (C or CS) on female mice

Female KOs were influenced by both, genotype and condition with respect to emotion-like behaviour in the present study. They displayed reduced depression-like behavior (consistently in both, the SI and TS test), in both comparisons, CS vs. C (condition effect) and KO vs. WT after CS (genotype effect). This is in sharp contrast to published studies reporting increased depression-like behaviour (among others) after stress [6–9, 66]. Our female KOs appeared rather as behaviourally disinhibited and explorative to novel environments (behavioural test arenas) in response to CS. Assuming the above mentioned especially mild CS paradigm in our present study, female KOs appeared not only distinctly less stress-susceptible than female WTs but even emotionally CS-insensitive. In support of this assumption, we detected statistically unchanged brain fibres in female KOs after CS (vs. C) in contrast to decreased fibres in female WTs after CS (vs. C). Our female KOs might thus have hippocampi insensitive to CS, in contrast to the above mentioned and typically observed negative consequences of CS such as reduced neurogenesis, dendrites, pathways, connectivities and synaptic functions [64, 65].

### The potentially resilient response of *Zdhhc7* KO females to CS condition

Female KOs revealed upregulated *Cort* expression after CS vs. C in their hippocampi. *Cort* encodes the neuropeptide cortistatin from the somatostatin/urotensin family and is mainly expressed in cortical and hippocampal GABAergic interneurons [67]. Cortistatin is involved in regulating hypothalamic pituitary axis (HPA) mediated stress responses in mice [68, 69] and mice lacking cortistatin showed increased levels of stress-related glucocorticoids and anxiety-like behavior [70, 71]. In humans, reductions in cortistatin expression were associated with cognitive defects observed in Alzheimer’s disease patients [72] as well as with increased depression in patients with major depressive disorder [73, 74]. Upregulation of cortistatin in our female KOs in the present study might thus have led to the observed slightly better cognitive performance and the significantly reduced depression-like behavior after CS. In support for this, Jiang and colleagues [75] reported antidepressant-like effects in mice after centrally administered cortistatin. In our mice, the underlying mechanism might have involved a form of KO-linked cortistatin-triggered brain microstructural reorganisation so that neither CS-induced fibre impairment occurred (as compared to female WTs) nor KO-induced fibre impairment (as described for control condition by Kerkenberg et al. [61]). In line with this, Gonzalez-Rey et al. [71] reviewed that treatment with cortistatin in mouse models of multiple sclerosis could partly recover brain pathology, maybe by increasing neurotrophic and neuroprotective proteins critical in repair and regeneration maybe by the induction of axonal outgrowth and remyelination. Our method of DTI-based fibre tractography might have indeed identified such improved axonal outgrowth and remyelination based on upregulated *Cort* and dependent on *Zdhhc7*-deficiency and CS at least in females.

### Comparison of findings in *Zdhhc7* females with findings in *Zdhhc7* males

In the present study, males showed few condition effects (CS vs. C), including only the behavioural level. Both WTs and KOs increased their locomotion after CS similar to females and in line to other studies [8, 63]. Increased locomotion was accompanied by better attention and lower stress load without changes of emotionality and brain microstructural or gene expression changes. These findings are in contrast to previous findings after stress conditions [6–9, 66] and might indicate a more active, uninhibited and exploratory coping style in response to CS in our males. Our aforementioned presumably very mild and only 4 weeks lasting CS paradigm could be responsible for this. Further, sex differences might account for the few findings in males, which are less prone to stress induced changes in anxiety- or depression-like behaviours in rodents and humans (e.g., [76–81]). Furthermore, particularly hippocampal neurons are sensitive to chronic stress in a sex-specific way [33, 64, 82, 83]. Since our investigated *Zdhhc7* mouse model is already described to reveal sex differences in the control condition [51] and after acute stress [52], it is plausible, that this should also be the case after CS. Such sex-specificity could be expected to be more pronounced after CS because of investigating Z*dhhc7* mice of an older age (15 weeks in present study) compared to younger Z*dhhc7* mice after acute stress (11 weeks in Kerkenberg et al. [52]). As mentioned above, younger Z*dhhc7* mice are still in the process of ongoing brain development [61] and thus differences between sexes as well as between genotypes should be more distinct in older mice like in our present study.

## Conclusions

Taken together, our mild and comparably short CS paradigm might have led to the overall few findings, which were mostly present in females. This is in line with the literature, reporting females to be more prone to stronger stress responses than males and with our hypothesis of a sex-specificity of stress responses in our *Zdhhc7* mouse model. Compared to female WTs our female KOs seemed to have been protected from CS with overall resilient responses on behavioural, brain microstructural and gene expression levels. The reason for this could be *Cort*, whose upregulation by CS might have led to neuroprotective effects, a reorganisation and regeneration of hippocampal fibres and thus possibly also a particular antidepressant effect on the level of behaviour. A *Zdhhc7*-deficiency dependent sex-specific hippocampal organisation could possibly prepare the basis for providing a kind of protection against at least mild CS via *Cort*. Or, to put it another way, the combination of female sex and the absence of *Zdhhc7* may be what enables an upregulation of *Cort* and an insensitivity or even resilience to mild CS.

### Supplementary materials

Supplementary materials are provided in the form of Suppl. Tab. 1 (Behavioural analyses with statistical test details), Suppl. Tab. 2 (Brain microstructure analyses with statistical test details) and Suppl. Tab. 3 (Gene expression analyses with statistical test details). Further materials are provided in the form of Suppl. Fig. 1 (Timeline of behavioural testing), Suppl. Fig. 2 (Heatmap and dendrogram illustrating hippocampal genes in different group comparisons), Suppl. Fig. 3 (Heatmap and dendrogram illustrating prefrontal cortex genes in different group comparisons), Suppl. Fig. 4 (Heatmap and dendrogram illustrating hippocampal (Fig. A) and prefrontal cortex (Fig. B) gene counts sample by sample), Suppl. Fig. 5 (Volcanoplots illustrating hippocampal genes in different group comparisons) and Suppl. Fig. 6 (Volcanoplots illustrating prefrontal cortex genes in different group comparisons).

## Supplementary information

Supplemental Material

Supplemental Figures

## Data Availability

All raw data supporting the findings of this study are available from the corresponding author upon reasonable request.
