# Supplementary material for: Deficiency of the palmitoyl acyltransferase ZDHHC7 modulates depression-like behaviour in female mice after a mild chronic stress paradigm
Source: Transl Psychiatry. 2025 Jan 24;15:20. doi: 10.1038/s41398-025-03240-7 (PMC11759705; doi:10.1038/s41398-025-03240-7)
Supplement: Supplementary file 1 — Supplemental Material [file 41398_2025_3240_MOESM1_ESM.docx]

Supplemental Tab. 1: Behavioural analyses with statistical test details (Mann-Whitney U test)

| **Test, parameter, effect** | **Groups tested** | ***U* value** | ***z* value** | ***p* value** |
| --- | --- | --- | --- | --- |
| SAT, distance [m], genotype effects | WT_f_C vs. KO_f_C | 159.0 | -2.660 | 0.008 ** |
|  | WT_f_CS vs. KO_f_CS | 357.5 | 2.043 | 0.041 * |
|  | WT_m_C vs. KO_m_C | 182.0 | -0.968 | 0.333 |
|  | WT_m_CS vs. KO_m_CS | 264.0 | 0.516 | 0.606 |
| SAT, alternation score [%], genotype effects | WT_f_C vs. KO_f_C | 358.0 | 1.444 | 0.149 |
|  | WT_f_CS vs. KO_f_CS | 345.0 | 1.769 | 0.077 (T) |
|  | WT_m_C vs. KO_m_C | 209.0 | -0.290 | 0.772 |
|  | WT_m_CS vs. KO_m_CS | 272.0 | 0.705 | 0.481 |
| EPM, distance [m], genotype effects | WT_f_C vs. KO_f_C | 235.0 | -0.648 | 0.517 |
|  | WT_f_CS vs. KO_f_CS | 346.0 | 1.790 | 0.073 (T) |
|  | WT_m_C vs. KO_m_C | 169.0 | -1.296 | 0.195 |
|  | WT_m_CS vs. KO_m_CS | 285.0 | 1.009 | 0.313 |
| EPM, relative time spent on open arms [%],  genotype effects | WT_f_C vs. KO_f_C | 246.0 | -0.406 | 0.684 |
|  | WT_f_CS vs. KO_f_CS | 305.0 | 0.890 | 0.374 |
|  | WT_m_C vs. KO_m_C | 243.0 | 0.566 | 0.571 |
|  | WT_m_CS vs. KO_m_CS | 274.0 | 0.751 | 0.453 |
| SI, social interaction ratio, genotype effects | WT_f_C vs. KO_f_C | 267.0 | -0.433 | 0.665 |
|  | WT_f_CS vs. KO_f_CS | 382.0 | 2.581 | 0.010 ** |
|  | WT_m_C vs. KO_m_C | 218.0 | -0.563 | 0.573 |
|  | WT_m_CS vs. KO_m_CS | 197.0 | -0.591 | 0.554 |
| TS, immobility time [s] , genotype effects | WT_f_C vs. KO_f_C | 284.0 | -0.082 | 0.934 |
|  | WT_f_CS vs. KO_f_CS | 163.0 | -2.236 | 0.025 * |
|  | WT_m_C vs. KO_m_C | 178.0 | -1.502 | 0.133 |
|  | WT_m_CS vs. KO_m_CS | 244.0 | 0.047 | 0.963 |
| NB, score after 1 h, genotype effects | WT_f_C vs. KO_f_C | 288.0 | <0.1 | 0.999 |
|  | WT_f_CS vs. KO_f_CS | 276.0 | 1.0 | 0.317 |
|  | WT_m_C vs. KO_m_C | 286.0 | 1.546 | 0.122 |
|  | WT_m_CS vs. KO_m_CS | 253.0 | 1.0 | 0.317 |
| NB, score after 3h, genotype effects | WT_f_C vs. KO_f_C | 314.5 | 0.614 | 0.539 |
|  | WT_f_CS vs. KO_f_CS | 241.5 | -0.664 | 0.507 |
|  | WT_m_C vs. KO_m_C | 265.0 | 0.593 | 0.554 |
|  | WT_m_CS vs. KO_m_CS | 267.5 | 0.633 | 0.527 |
| NB, score after 7h, genotype effects | WT_f_C vs. KO_f_C | 295.5 | 0.182 | 0.855 |
|  | WT_f_CS vs. KO_f_CS | 273.5 | 0.213 | 0.831 |
|  | WT_m_C vs. KO_m_C | 242.0 | <0.1 | 0.999 |
|  | WT_m_CS vs. KO_m_CS | 248.0 | 0.151 | 0.880 |
| NB, score after 24 h, genotype effects | WT_f_C vs. KO_f_C | 336.0 | 1.073 | 0.283 |
|  | WT_f_CS vs. KO_f_CS | 329.5 | 1.478 | 0.139 |
|  | WT_m_C vs. KO_m_C | 240.5 | -0.037 | 0.971 |
|  | WT_m_CS vs. KO_m_CS | 289.5 | 1.286 | 0.198 |

To be continued

Supplemental Tab. 1: continued

| SAT, distance [m], condition effects | WT_f_C vs. WT_f_CS | 408.0 | 2.809 | 0.005 ** |
| --- | --- | --- | --- | --- |
|  | KO_f_C vs. KO_f_CS | 540.0 | 5.618 | <0.001 *** |
|  | WT_m_C vs. WT_m_CS | 383.0 | 3.693 | <0.001 *** |
|  | KO_m_C vs. KO_m_CS | 423.0 | 4.665 | <0.001 *** |
| SAT, alternation score [%], condition effects | WT_f_C vs. WT_f_CS | 295.5 | 0.415 | 0.678 |
|  | KO_f_C vs. KO_f_CS | 278.0 | 0.043 | 0.966 |
|  | WT_m_C vs. WT_m_CS | 154.0 | -1.872 | 0.061 (T) |
|  | KO_m_C vs. KO_m_CS | 194.0 | -0.900 | 0.368 |
| EPM, distance [m], condition effects | WT_f_C vs. WT_f_CS | 414.0 | 3.284 | 0.001 *** |
|  | KO_f_C vs. KO_f_CS | 509.0 | 5.371 | <0.001 *** |
|  | WT_m_C vs. WT_m_CS | 407.0 | 4.276 | <0.001 *** |
|  | KO_m_C vs. KO_m_CS | 435.0 | 4.956 | <0.001 *** |
| EPM, relative time spent on open arms [%], condition effects | WT_f_C vs. WT_f_CS | 186.0 | -1.726 | 0.084 (T) |
|  | KO_f_C vs. KO_f_CS | 254.0 | -0.231 | 0.818 |
|  | WT_m_C vs. WT_m_CS | 227.0 | -0.097 | 0.923 |
|  | KO_m_C vs. KO_m_CS | 229.0 | -0.049 | 0.961 |
| SI, social interaction ratio, condition effects | WT_f_C vs. WT_f_CS | 267.0 | -0.192 | 0.848 |
|  | KO_f_C vs. KO_f_CS | 396.0 | 2.554 | 0.011 * |
|  | WT_m_C vs. WT_m_CS | 247.0 | 0.389 | 0.697 |
|  | KO_m_C vs. KO_m_CS | 275.0 | 1.069 | 0.285 |
| TS, immobility time [s] , condition effects | WT_f_C vs. WT_f_CS | 233.0 | -0.915 | 0.360 |
|  | KO_f_C vs. KO_f_CS | 143.5 | -2.827 | 0.005 ** |
|  | WT_m_C vs. WT_m_CS | 204.5 | -0.880 | 0.379 |
|  | KO_m_C vs. KO_m_CS | 281.0 | 0.916 | 0.360 |
| NB, score after 1 h, condition effects | WT_f_C vs. WT_f_CS | 253.0 | -1.400 | 0.162 |
|  | KO_f_C vs. KO_f_CS | 265.0 | -0.553 | 0.580 |
|  | WT_m_C vs. WT_m_CS | 220.0 | -1.431 | 0.152 |
|  | KO_m_C vs. KO_m_CS | 187.0 | -2.037 | 0.042 * |
| NB, score after 3h, condition effects | WT_f_C vs. WT_f_CS | 215.0 | -1.508 | 0.132 |
|  | KO_f_C vs. KO_f_CS | 161.0 | -2.836 | 0.005 ** |
|  | WT_m_C vs. WT_m_CS | 266.5 | 0.617 | 0.537 |
|  | KO_m_C vs. KO_m_CS | 266.0 | 0.591 | 0.554 |
| NB, score after 7h, condition effects | WT_f_C vs. WT_f_CS | 339.5 | 1.518 | 0.129 |
|  | KO_f_C vs. KO_f_CS | 340.5 | 1.522 | 0.128 |
|  | WT_m_C vs. WT_m_CS | 358.0 | 2.843 | 0.004 ** |
|  | KO_m_C vs. KO_m_CS | 342.5 | 2.472 | 0.013 * |
| NB, score after 24 h, condition effects | WT_f_C vs. WT_f_CS | 337.0 | 1.377 | 0.169 |
|  | KO_f_C vs. KO_f_CS | 355.0 | 1.756 | 0.080 (T) |
|  | WT_m_C vs. WT_m_CS | 298.5 | 1.416 | 0.157 |
|  | KO_m_C vs. KO_m_CS | 337.5 | 2.471 | 0.013 * |

WT: *Zdhhc7* wildtype, KO: *Zdhhc7* knockout, f: female, m: male, C: control condition, CS: chronic stress condition, SAT: spontaneous alternation test, EPM: elevated plus maze, SI: social interaction test, TS: tail suspension test, m: meter, s: seconds, h: hours; sample sizes: n=22-24 per group; (T): p<0.10; *: p<0.05; **: p<0.01; ***: p<0.001.

Supplemental Tab. 2: Brain microstructure analyses with statistical test details (Mann-Whitney U test)

| **Brain region, parameter, effect** | **Groups tested** | ***U* value** | ***z* value** | ***p* value** |
| --- | --- | --- | --- | --- |
| Hippocampus (ventral, left),  fibre number,  genotype effects | KO_f_C vs. WT_f_C | 23.0 | 2.200 | 0.032 * |
|  | KO _f_CS vs. WT _f_CS | 4.0 | -1.155 | 0.343 |
|  | KO _m_C vs. WT _m_C | 2.5 | -1.597 | 0.114 |
|  | KO _m_CS vs. WT _m_CS | 4.0 | -1.155 | 0.343 |
| Hippocampus (ventral, left),  mean fibre length,  genotype effects | KO _f_C vs. WT _f_C | 23.0 | 2.193 | 0.032 * |
|  | KO _f_CS vs. WT _f_CS | 5.0 | -0.866 | 0.486 |
|  | KO _m_C vs. WT _m_C | 4.0 | -1.155 | 0.343 |
|  | KO _m_CS vs. WT _m_CS | 3.0 | -1.443 | 0.200 |
| Hippocampus (ventral, right),  fibre number,  genotype effects | KO _f_C vs. WT _f_C | 18.0 | 1.149 | 0.310 |
|  | KO _f_CS vs. WT _f_CS | 2.5 | -1.637 | 0.114 |
|  | KO _m_C vs. WT _m_C | 4.0 | -1.155 | 0.343 |
|  | KO _m_CS vs. WT _m_CS | 5.5 | -0.726 | 0.486 |
| Hippocampus (ventral, right), mean fibre length,  genotype effects | KO _f_C vs. WT _f_C | 16.0 | 0.731 | 0.548 |
|  | KO _f_CS vs. WT _f_CS | 7.0 | -0.289 | 0.886 |
|  | KO _m_C vs. WT _m_C | 3.0 | -1.443 | 0.200 |
|  | KO _m_CS vs. WT _m_CS | 7.0 | -0.289 | 0.886 |
| Hippocampus (ventral, left),  fibre number,  condition effects | WT_f_C vs. WT_f_CS | 1.0 | -2.214 | 0.032 * |
|  | KO_f_C vs. KO_f_CS | 14.0 | 0.980 | 0.413 |
|  | WT_m_C vs. WT_m_CS | 2.0 | -1.732 | 0.114 |
|  | KO_m_C vs. KO_m_CS | 6.0 | -0.577 | 0.686 |
| Hippocampus (ventral, left),  mean fibre length,  condition effects | WT_f_C vs. WT_f_CS | 6.0 | -0.980 | 0.413 |
|  | KO_f_C vs. KO_f_CS | 15.0 | 1.225 | 0.286 |
|  | WT_m_C vs. WT_m_CS | 2.0 | -1.732 | 0.114 |
|  | KO_m_C vs. KO_m_CS | 6.0 | -0.577 | 0.686 |
| Hippocampus (ventral, right),  fibre number,  condition effects | WT_f_C vs. WT_f_CS | 3.0 | -1.722 | 0.111 |
|  | KO_f_C vs. KO_f_CS | 9.0 | -0.245 | 0.905 |
|  | WT_m_C vs. WT_m_CS | 8.0 | 0.000 | 0.999 |
|  | KO_m_C vs. KO_m_CS | 6.0 | -0.577 | 0.686 |
| Hippocampus (ventral, right), mean fibre length,  condition effects | WT_f_C vs. WT_f_CS | 1.0 | -2.205 | 0.032 * |
|  | KO_f_C vs. KO_f_CS | 3.0 | -1.715 | 0.111 |
|  | WT_m_C vs. WT_m_CS | 13.0 | 1.443 | 0.200 |
|  | KO_m_C vs. KO_m_CS | 9.0 | 0.289 | 0.999 |

WT: *Zdhhc7* wildtype, KO: *Zdhhc7* knockout, f: female, m: male, C: control condition, CS: chronic stress condition; sample sizes: n=4-5 per group; *: p<0.05.

Supplemental Tab. 3: Gene expression analyses with statistical test details (logarithm of fold change, Wald statistics and adjusted false discovery rate)

| **Brain region, effect** | **Groups A vs. B** | **Ensembl gene ID** | **Gene symbol** | **Gene name** | **Log2-FC** | **Wald stat.** | ***p* value** | ***p* adj.** |
| --- | --- | --- | --- | --- | --- | --- | --- | --- |
| Hippocampus (ventral),  genotype effects | KO_f_C vs. WT_f_C | ENSMUSG00000031823 | *Zdhhc7* | zinc finger, DHHC domain containing 7 | -0.630 | -9.046 | <0.001 | <0.001 |
|  |  | ENSMUSG00000110631 | *Gm42047* | predicted gene, 42047 | -6.351 | -6.846 | <0.001 | <0.001 |
|  |  | ENSMUSG00000015013 | *Trappc2l* | trafficking protein particle complex 2-like | 0.326 | 5.183 | <0.001 | 0.002 |
|  |  | ENSMUSG00000006589 | *Aprt* | adenine phosphoribosyl transferase | -0.322 | -4.443 | <0.001 | 0.047 |
|  |  | ENSMUSG00000019478 | *Rab4a* | RAB4A, member RAS oncogene family | 0.283 | 4.408 | <0.001 | 0.047 |
|  |  | ENSMUSG00000068196 | *Col8a1* | collagen, type VIII, alpha 1 | 2.756 | 4.354 | <0.001 | 0.050 |
|  | KO_f_CS vs. WT_f_CS | ENSMUSG00000031823 | *Zdhhc7* | zinc finger, DHHC domain containing 7 | -0.641 | -9.216 | <0.001 | <0.001 |
|  |  | ENSMUSG00000110631 | *Gm42047* | predicted gene, 42047 | -6.113 | -6.575 | <0.001 | <0.001 |
|  |  | ENSMUSG00000015013 | *Trappc2l* | trafficking protein particle complex 2-like | 0.357 | 5.692 | <0.001 | <0.001 |
|  |  | ENSMUSG00000019478 | *Rab4a* | RAB4A, member RAS oncogene family | 0.309 | 4.838 | <0.001 | 0.013 |
|  |  | ENSMUSG00000000738 | *Spg7* | SPG7, paraplegin matrix AAA peptidase subunit | 0.288 | 4.519 | <0.001 | 0.048 |
|  | KO_m_C vs. WT_m_C | ENSMUSG00000031823 | *Zdhhc7* | zinc finger, DHHC domain containing 7 | -0.630 | -7.378 | <0.001 | <0.001 |
|  |  | ENSMUSG00000110631 | *Gm42047* | predicted gene, 42047 | -6.137 | -5.388 | <0.001 | 0.001 |
|  | KO_m_CS vs. WT_m_CS | ENSMUSG00000031823 | *Zdhhc7* | zinc finger, DHHC domain containing 7 | -0.560 | -6,538 | <0.001 | <0.001 |
|  |  | ENSMUSG00000110631 | *Gm42047* | predicted gene, 42047 | -5.998 | -5,262 | <0.001 | 0.003 |
|  |  | ENSMUSG00000015013 | *Trappc2l* | trafficking protein particle complex 2-like | 0.372 | 4,805 | <0.001 | 0.020 |
| Hippocampus (ventral),  condition effects | WT_f_CS vs. WT_f_C | ENSMUSG00000066362 | *Rps13-ps1* | ribosomal protein S13, pseudogene 1 | -5.124 | -4.956 | <0.001 | 0.028 |
|  | KO_f_CS vs. KO_f_C | ENSMUSG00000028971 | *Cort* | cortistatin | 0.914 | 5.138 | <0.001 | 0.011 |
|  |  | ENSMUSG00000026579 | *F5* | coagulation factor V | -3.451 | -4.819 | <0.001 | 0.028 |
|  |  | ENSMUSG00000066362 | *Rps13-ps1* | ribosomal protein S13, pseudogene 1 | -4.836 | -4.672 | <0.001 | 0.038 |
|  | WT_m_CS vs. WT_m_C | - | **-** | - | - | - | - | n.s. |
|  | KO_m_CS vs. KO_m_C | - | - | - | - | - | - | n.s. |

To be continued

Supplemental Tab. 3: continued

| **Brain region, effect** | **Groups A vs. B** | **Ensembl gene ID** | **Gene symbol** | **Gene name** | **Log2-FC** | **Wald stat.** | ***p* value** | ***p* adj.** |
| --- | --- | --- | --- | --- | --- | --- | --- | --- |
| Medial prefrontal cortex,  genotype effects | KO_f_C vs. WT_f_C | ENSMUSG00000031823 | *Zdhhc7* | zinc finger, DHHC domain containing 7 | -0.668 | -9.025 | <0.001 | <0.001 |
|  |  | ENSMUSG00000110631 | *Gm42047* | predicted gene, 42047 | -6.969 | -7.714 | <0.001 | <0.001 |
|  | KO_f_CS vs. WT_f_CS | ENSMUSG00000031823 | *Zdhhc7* | zinc finger, DHHC domain containing 7 | -0.727 | -9.783 | <0.001 | <0.001 |
|  |  | ENSMUSG00000110631 | *Gm42047* | predicted gene, 42047 | -7.026 | -7.779 | <0.001 | <0.001 |
|  | KO_m_C vs. WT_m_C | ENSMUSG00000031823 | *Zdhhc7* | zinc finger, DHHC domain containing 7 | -0.646 | -7.157 | <0.001 | <0.001 |
|  |  | ENSMUSG00000110631 | *Gm42047* | predicted gene, 42047 | -6.995 | -6.323 | <0.001 | <0.001 |
|  |  | ENSMUSG00000049685 | *Cyp2g1* | cytochrome P450, family 2, subfamily g, polypeptide 1 | -16.358 | -6.262 | <0.001 | <0.001 |
|  |  | ENSMUSG00000019478 | *Rab4a* | RAB4A, member RAS oncogene family | 0.355 | 5.115 | <0.001 | 0.003 |
|  | KO_m_CS vs. WT_m_CS | ENSMUSG00000031823 | *Zdhhc7* | zinc finger, DHHC domain containing 7 | -0.723 | -8.029 | <0.001 | <0.001 |
|  |  | ENSMUSG00000110631 | *Gm42047* | predicted gene, 42047 | -6.117 | -5.525 | <0.001 | 0.001 |
| Medial prefrontal cortex,  condition effects | WT_f_CS vs. WT_f_C | ENSMUSG00000066362 | *Rps13-ps1* | ribosomal protein S13, pseudogene 1 | -5.138 | -5.262 | <0.001 | 0.005 |
|  | KO_f_CS vs. KO_f_C | ENSMUSG00000066362 | *Rps13-ps1* | ribosomal protein S13, pseudogene 1 | -4.9972 | -5.098 | <0.001 | 0.0126 |
|  | WT_m_CS vs. WT_m_C | - | - | - | - | - | - | n.s. |
|  | KO_m_CS vs. KO_m_C | ENSMUSG00000049685 | *Cyp2g1* | cytochrome P450, family 2, subfamily g, polypeptide 1 | 15.341 | 5.871 | <0.001 | <0.001 |

WT: *Zdhhc7* wildtype, KO: *Zdhhc7* knockout, f: female, m: male, C: control condition, CS: chronic stress condition, Log2-FC: logarithm (base of 2) of fold change, Wald stat.: Wald statistical test, p adj.: false discovery rate (FDR) adjusted p-value; sample sizes: n=4-6 per group.

Comparisons of group A vs. B: negative Log2-FC if gene expression in A is smaller, positive Log2-FC if gene expression in A is larger.
