# Supplementary material for: Deficiency of the palmitoyl acyltransferase ZDHHC7 modulates depression-like behaviour in female mice after a mild chronic stress paradigm
Source: Transl Psychiatry. 2025 Jan 24;15:20. doi: 10.1038/s41398-025-03240-7 (PMC11759705; doi:10.1038/s41398-025-03240-7)
Supplement: Supplementary file 2 — Supplemental Figures [file 41398_2025_3240_MOESM2_ESM.pdf]

Supplemental Figures

Suppl. Fig. 1

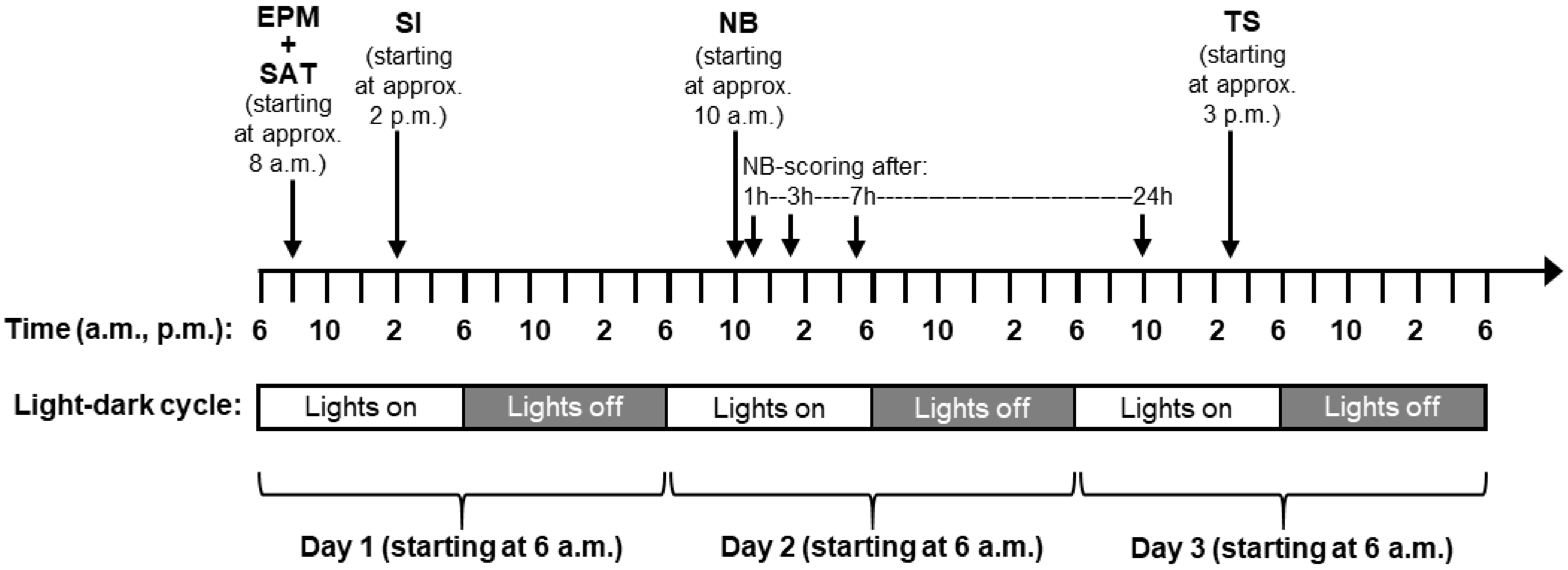

Supplemental Fig. 1 Timeline of behavioural testing. *Zdhhc7* wildtype and knockout mice (n=182) after control or chronic stress conditions at 14 weeks of age went through the following behavioural tests: elevated plus maze (EPM) test for the evaluation of anxiety-like behaviour and locomotion; spontaneous alternation test (SAT) for the evaluation of basal spatial working memory and locomotion; social interaction (SI) test for the assessment of depression-like behaviour; nest building (NB) test for the assessment of attention and general stress load; tail suspension (TS) test for the evaluation of depression-like behaviour.

Suppl. Fig. 2 A

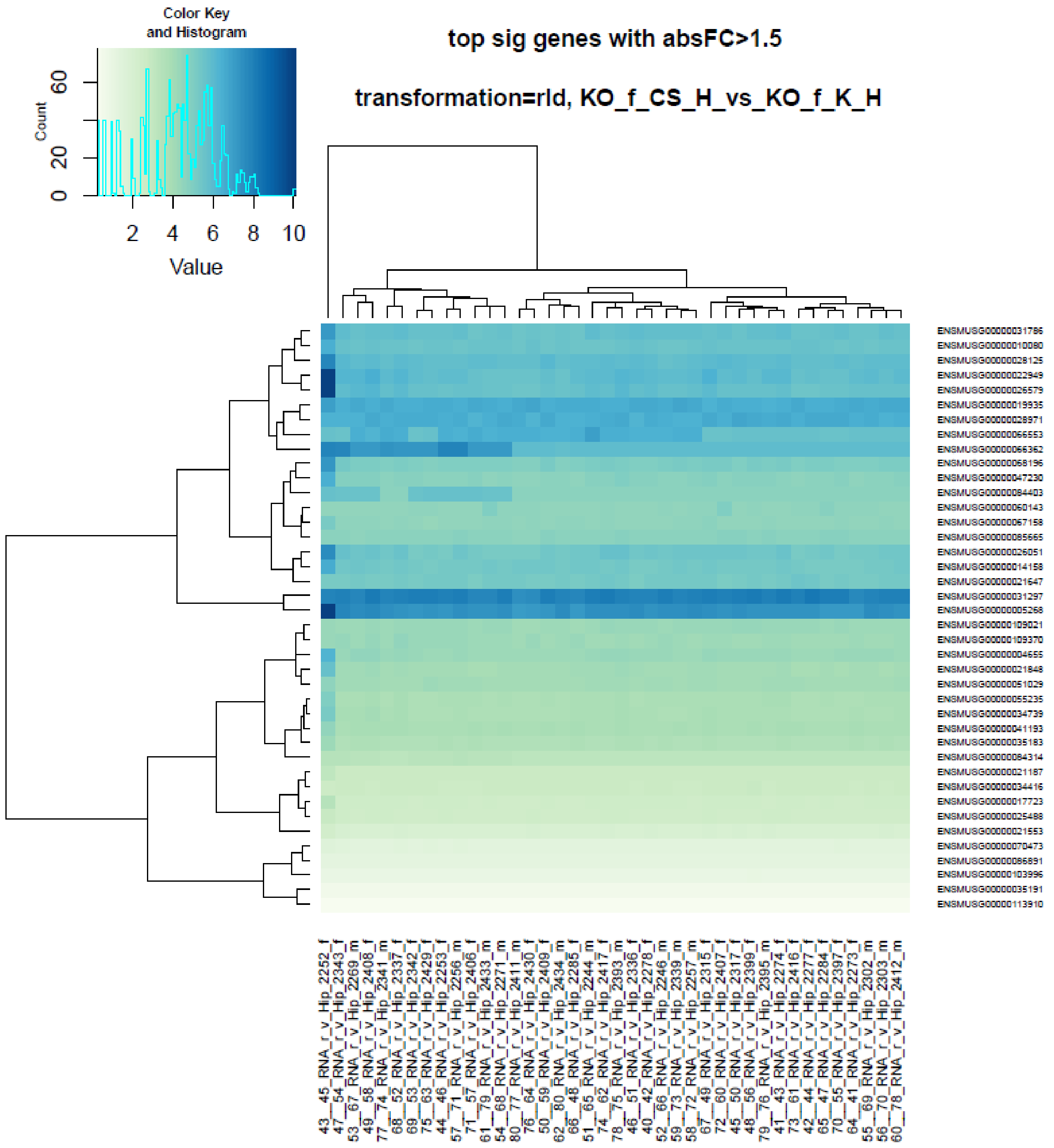

Suppl. Fig. 2 B

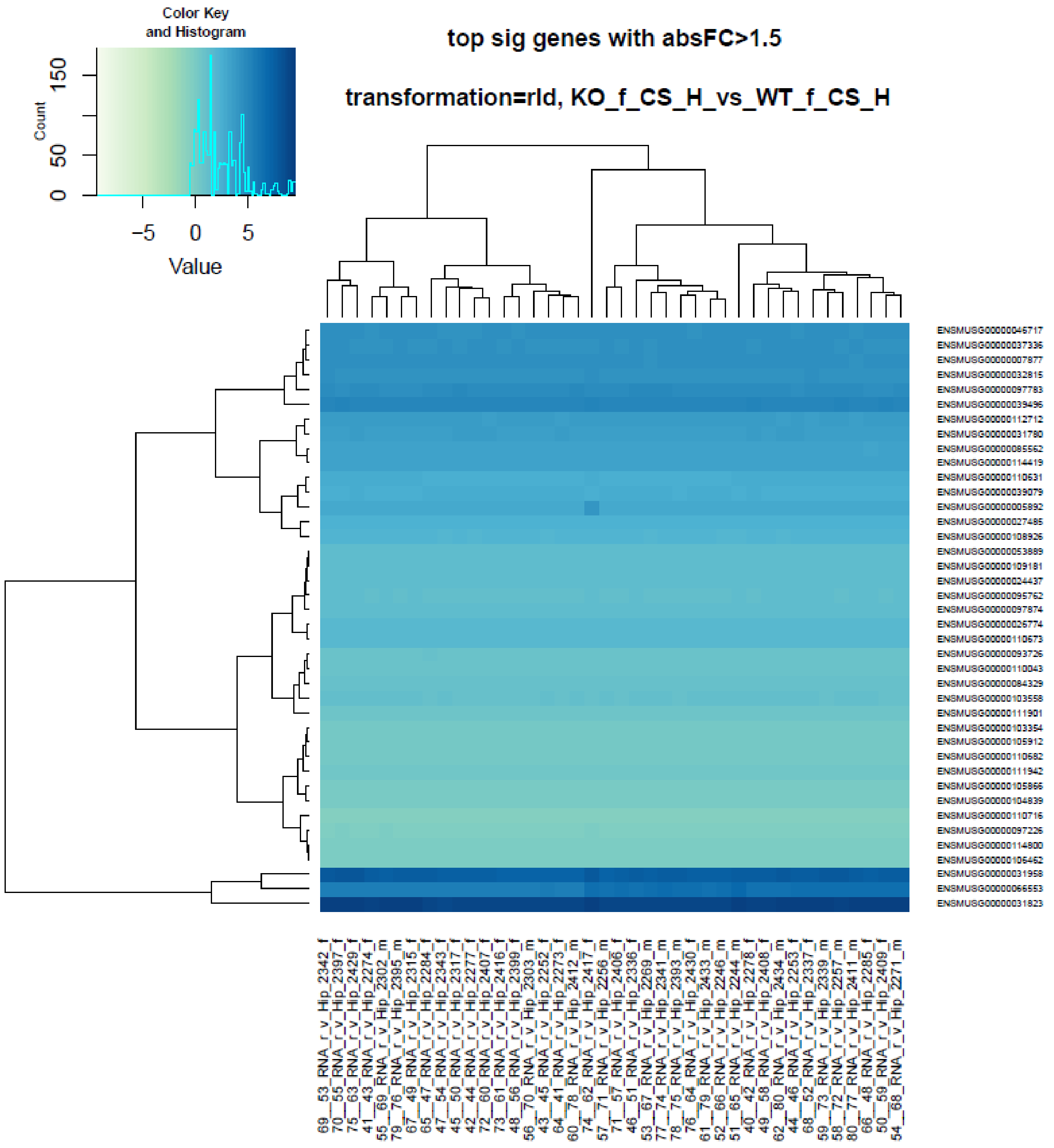

Supplemental Fig. 2 Heatmap and dendrogram illustrating hippocampal genes in different group comparisons: to be continued below

Suppl. Fig. 2 C

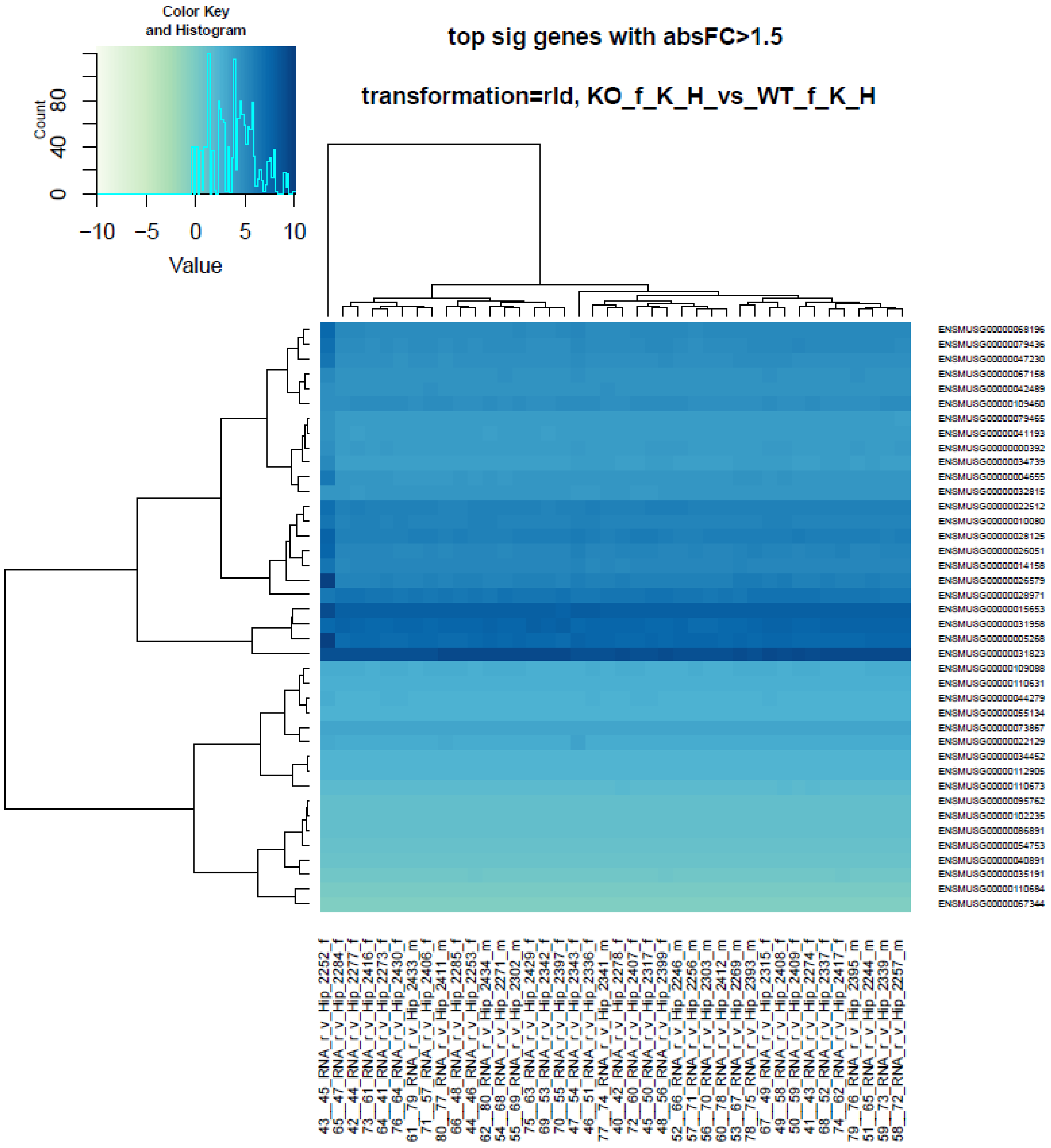

Suppl. Fig. 2 D

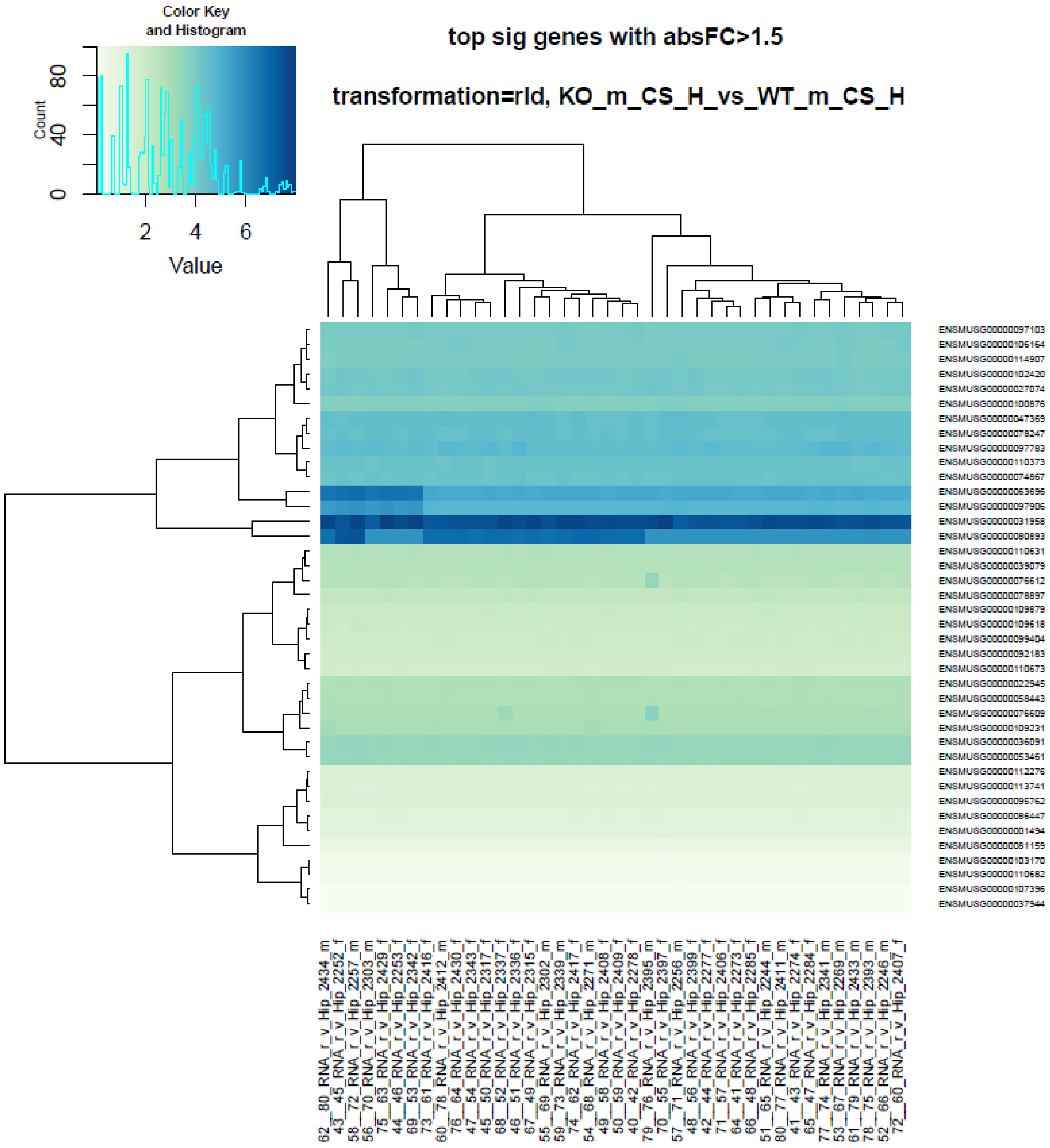

Supplemental Fig. 2 Heatmap and dendrogram illustrating hippocampal genes in different group comparisons: to be continued below

Suppl. Fig. 2 E

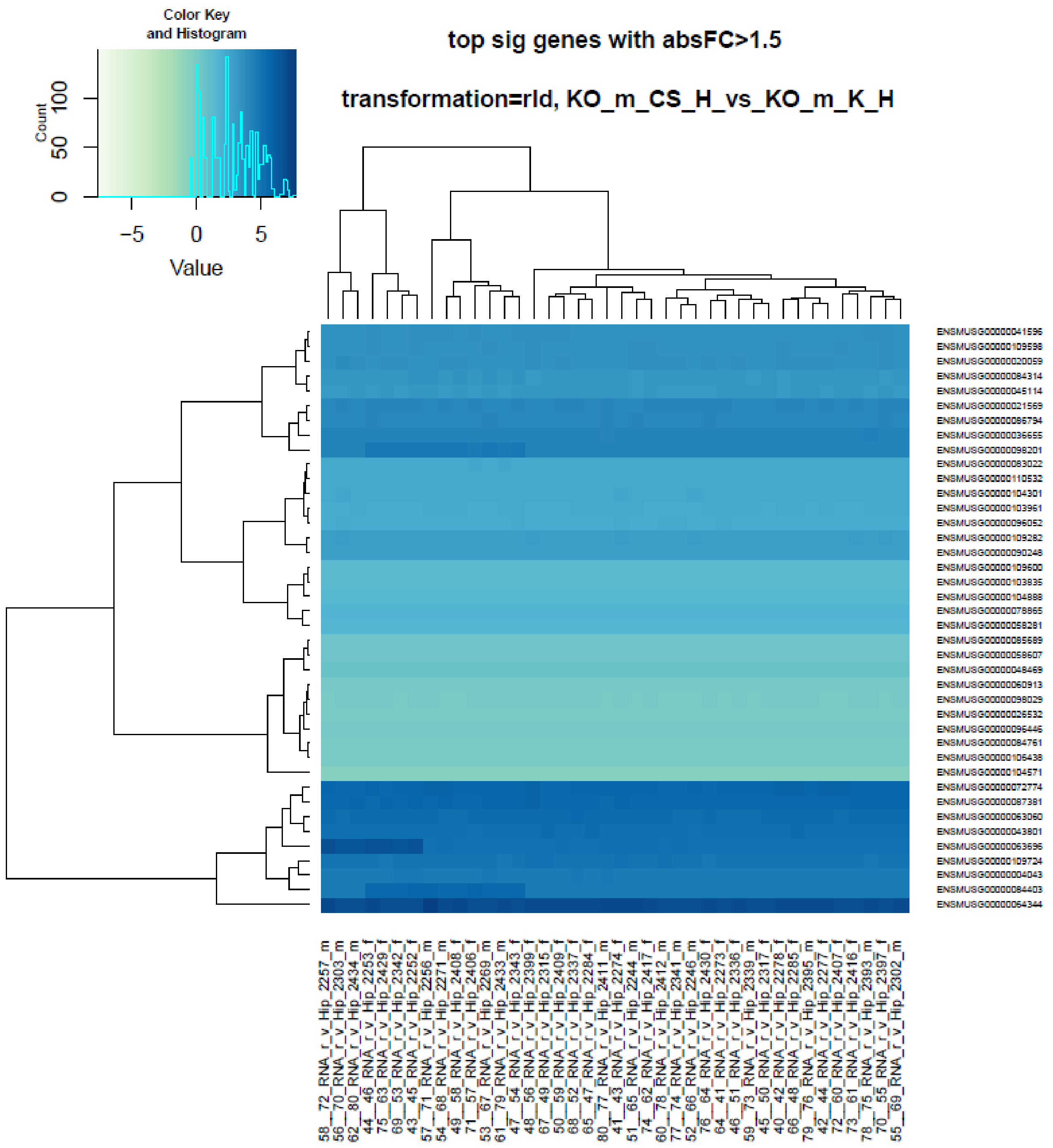

Suppl. Fig. 2 F

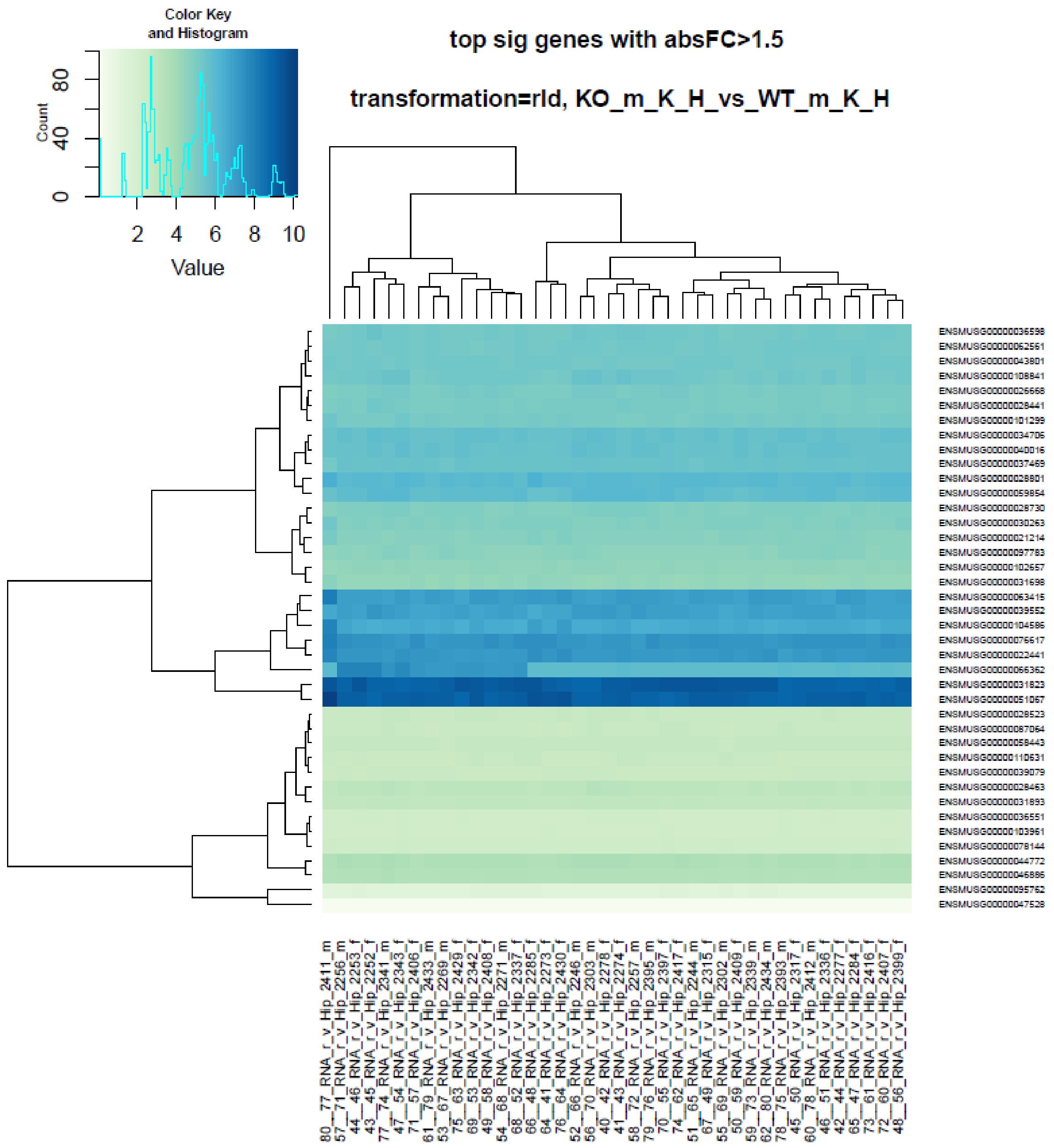

Supplemental Fig. 2 Heatmap and dendrogram illustrating hippocampal genes in different group comparisons: to be continued below

Suppl. Fig. 2 G

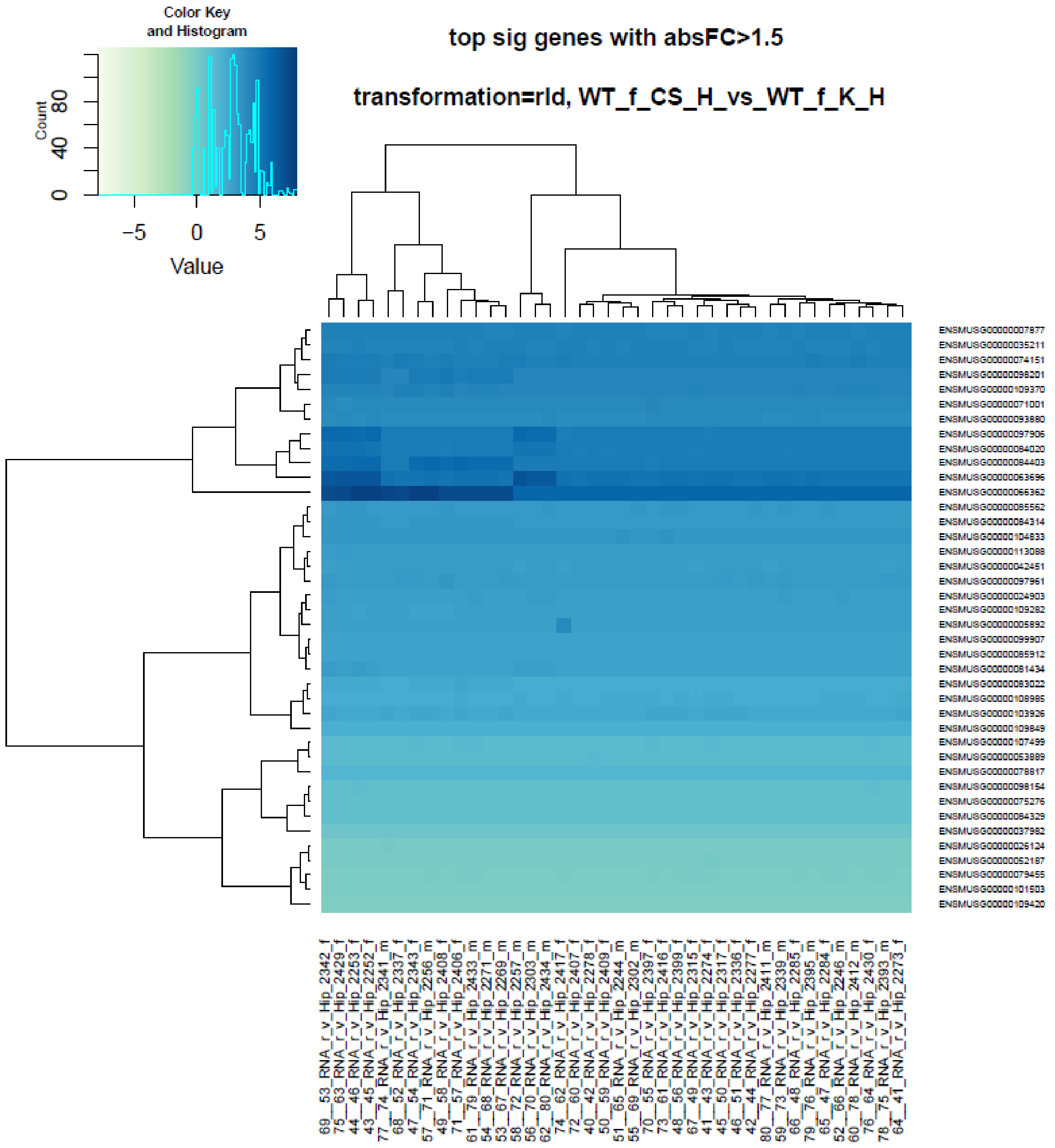

Suppl. Fig. 2 H

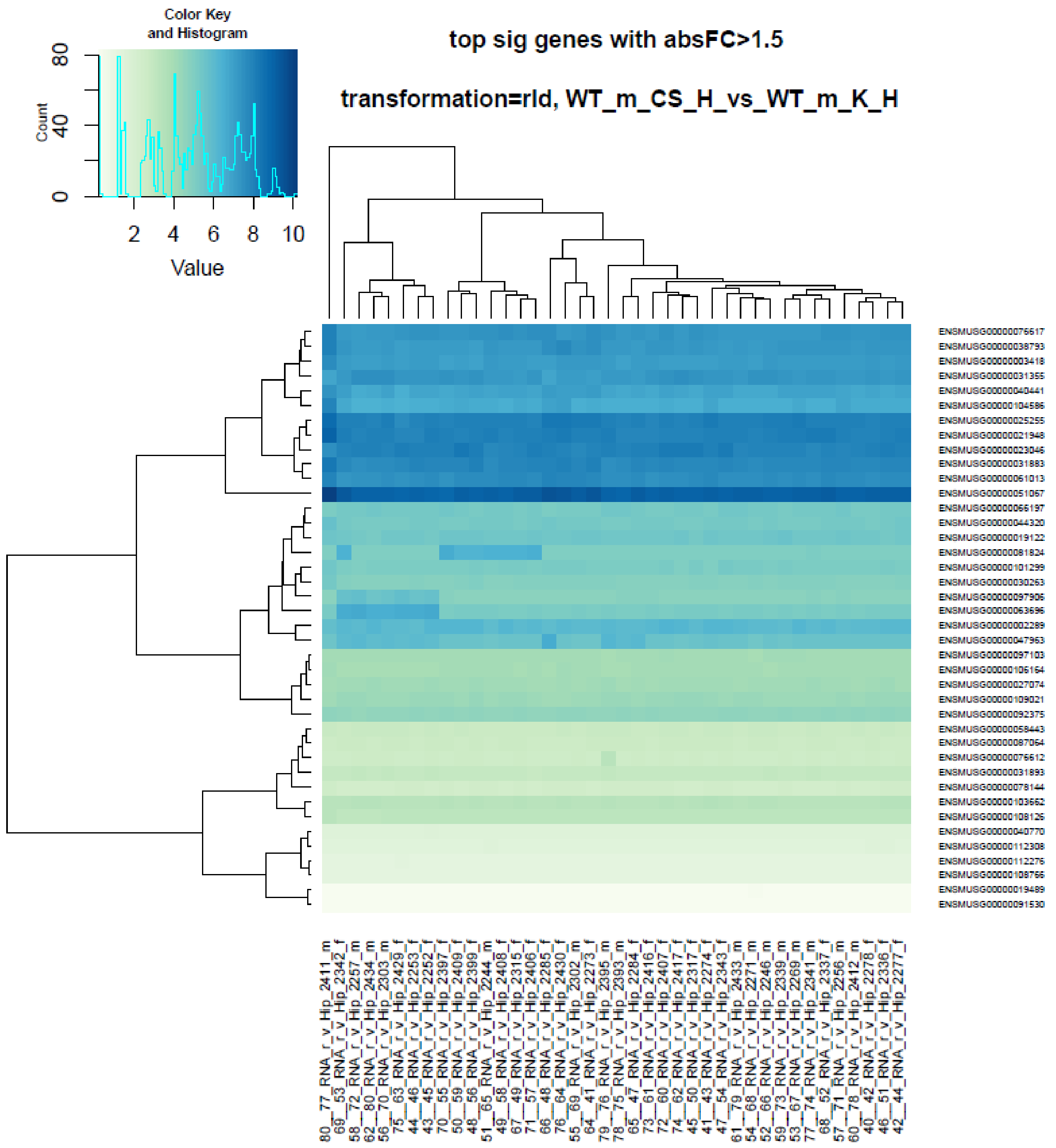

Supplemental Fig. 2 Heatmap and dendrogram illustrating hippocampal genes in different group comparisons. Top significant genes (ensemble IDs; statistics: Wald test) with absolute Fold changes (FC) > 1.5 were plotted against RNA-seq based gene expression data (normalized (rld transformation) counts) from *Zdhhc7* mice (N=40) from eight groups (1. C-WT-m, N=4; 2. CS-WT-m, N=4; 3. C-KO-m, N=4; 4. CS-KO-m, N=4; 5. C-WT-f, N=6; 6. CS-WT-f, N=6; 7. C-KO-f, N=6; 8. CS-KO-f N=6); for individual sample allocation to groups see GEO data deposition (GSE281404). Individual group comparisons were displayed in different parts of **Fig.: A)** KO_f_CS_H_vs._KO_f_K_H, **B)** KO_f_CS_H_vs._WT_f_CS_H, **C)** KO_f_K_H_vs._WT_f_K_H, **D)** KO_m_CS_H_vs._WT_m_CS_H, **E)** KO_m_CS_H_vs._KO_m_K_H, **F)** KO_m_K_H_vs._WT_m_K_H, **G)** WT_f_CS_H_vs._WT_f_K_H, **H)** WT_m_CS_H_vs._WT_m_K_H.

Suppl. Fig. 3 A

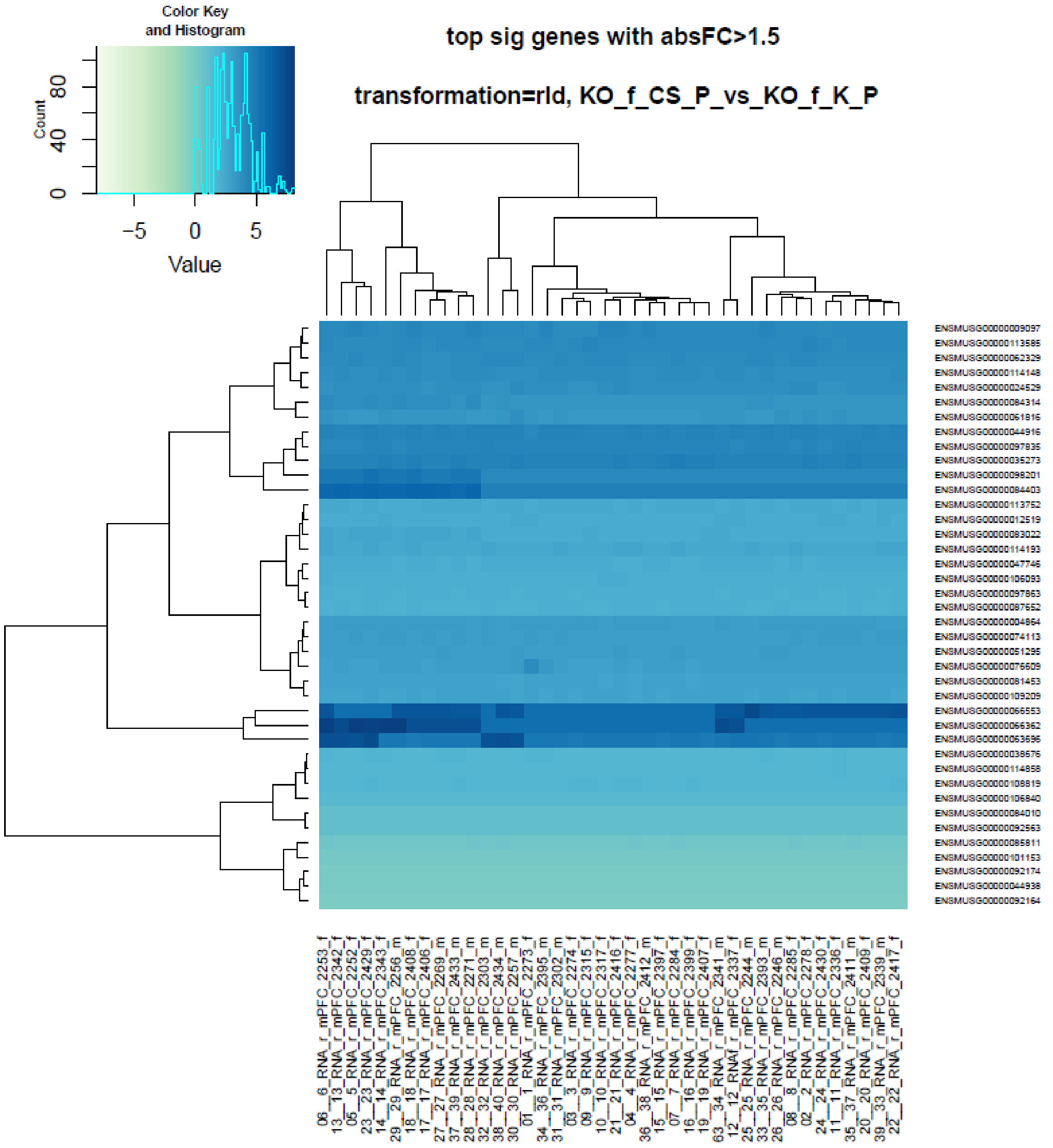

Suppl. Fig. 3 B

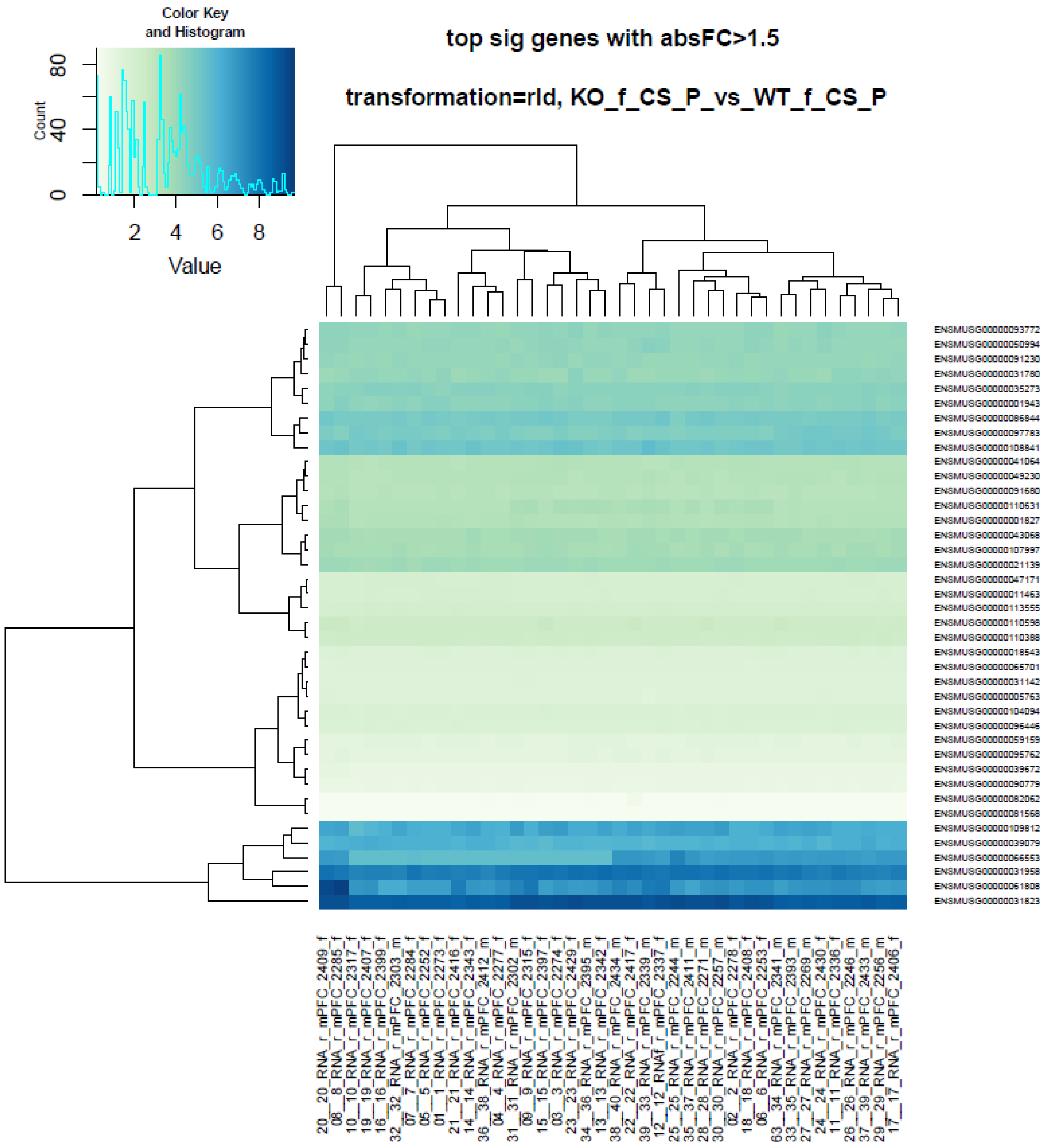

Supplemental Fig. 3 Heatmap and dendrogram illustrating prefrontal cortex genes in different group comparisons: to be continued below

Suppl. Fig. 3 C

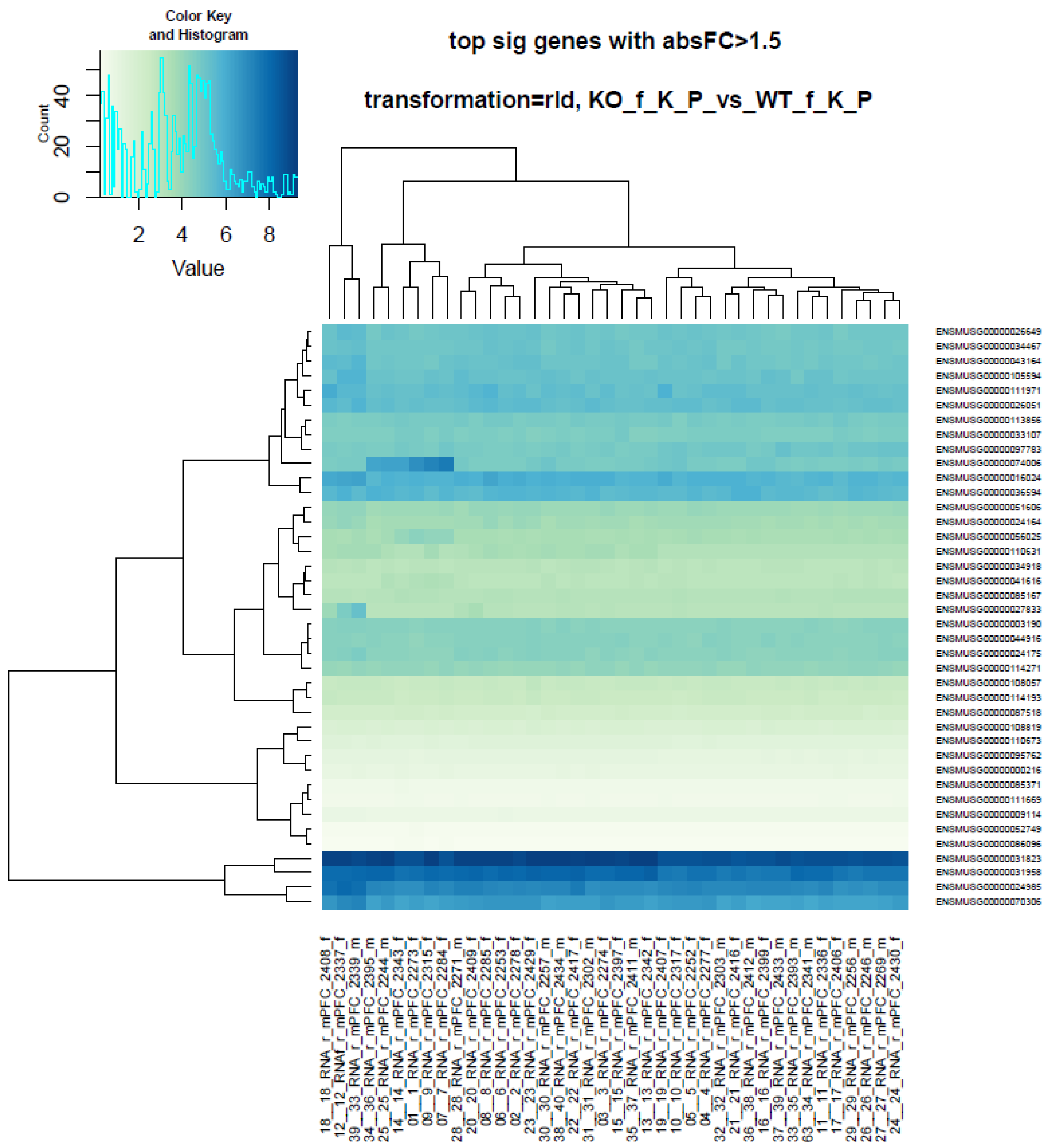

Suppl. Fig. 3 D

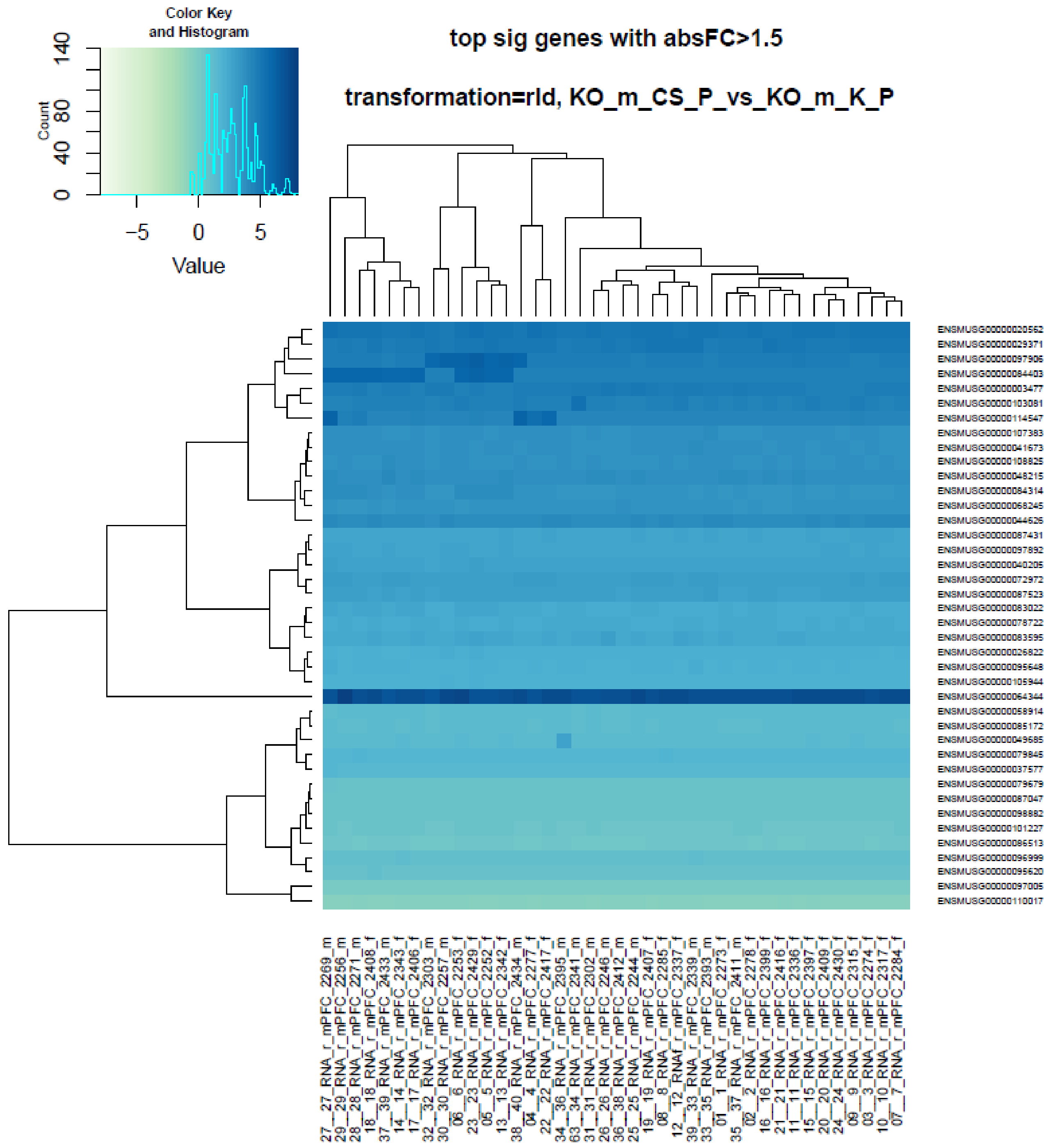

Supplemental Fig. 3 Heatmap and dendrogram illustrating prefrontal cortex genes in different group comparisons: to be continued below

Suppl. Fig. 3 E

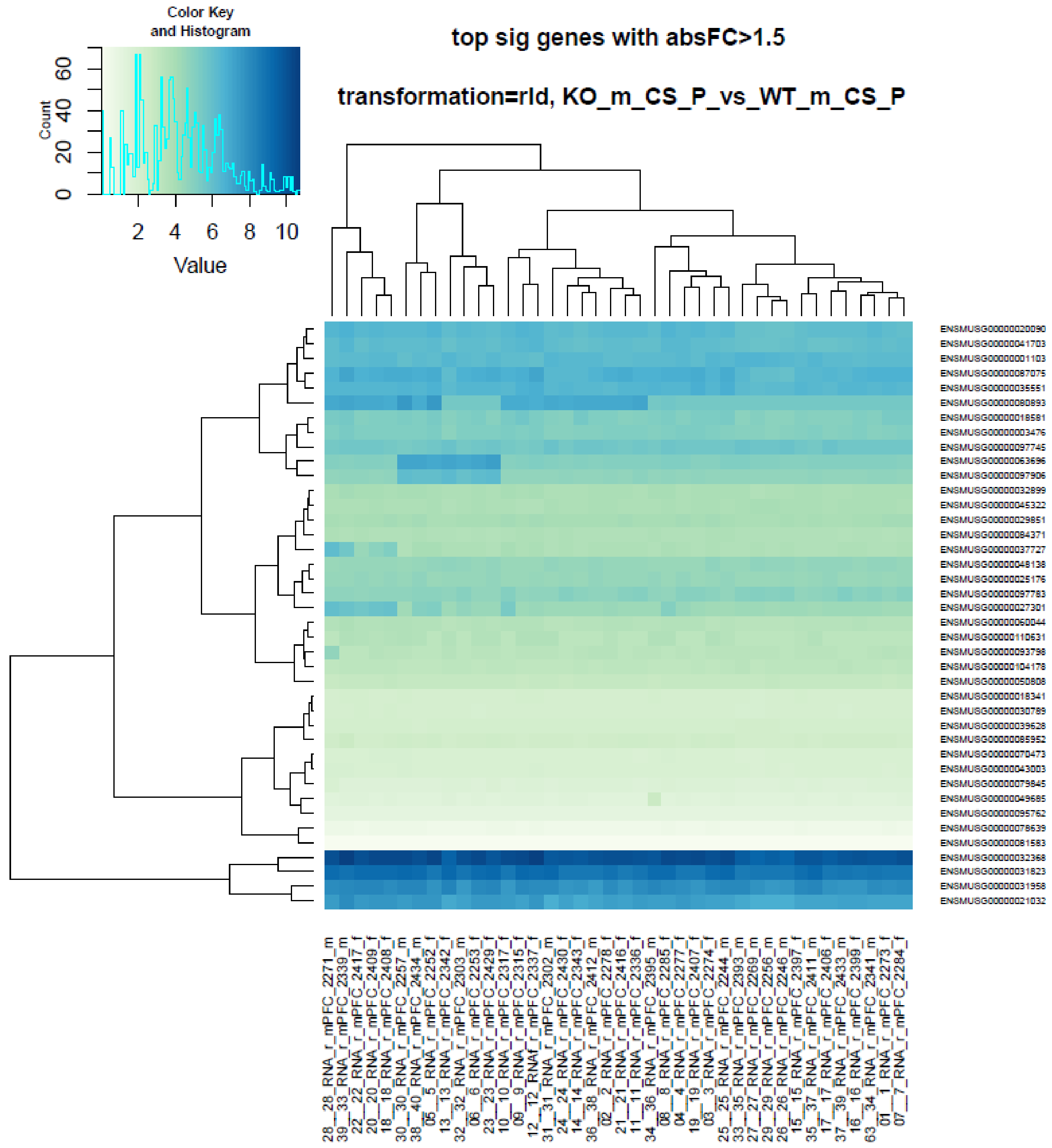

Suppl. Fig. 3 F

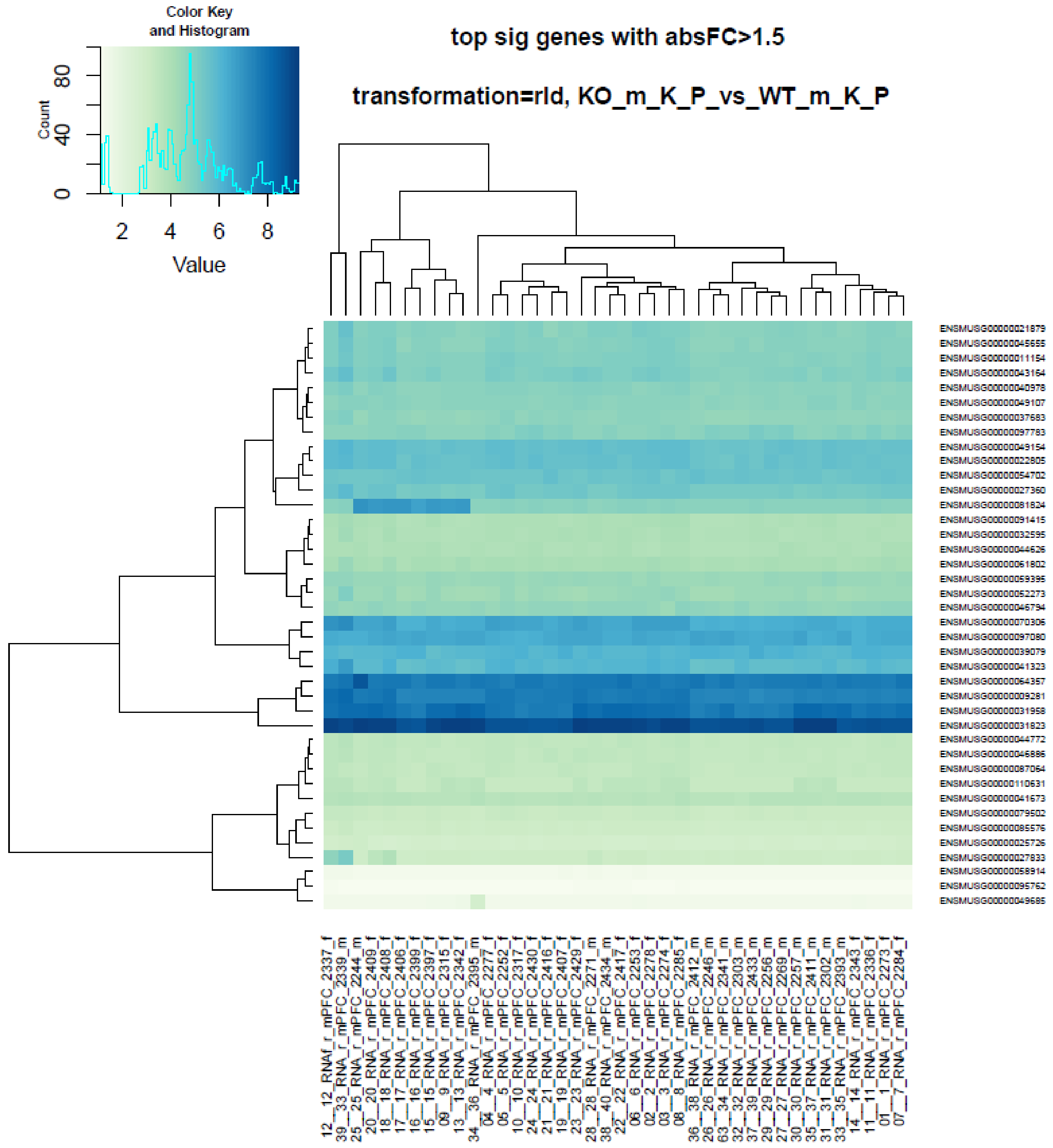

Supplemental Fig. 3 Heatmap and dendrogram illustrating prefrontal cortex genes in different group comparisons: to be continued below

Suppl. Fig. 3 G

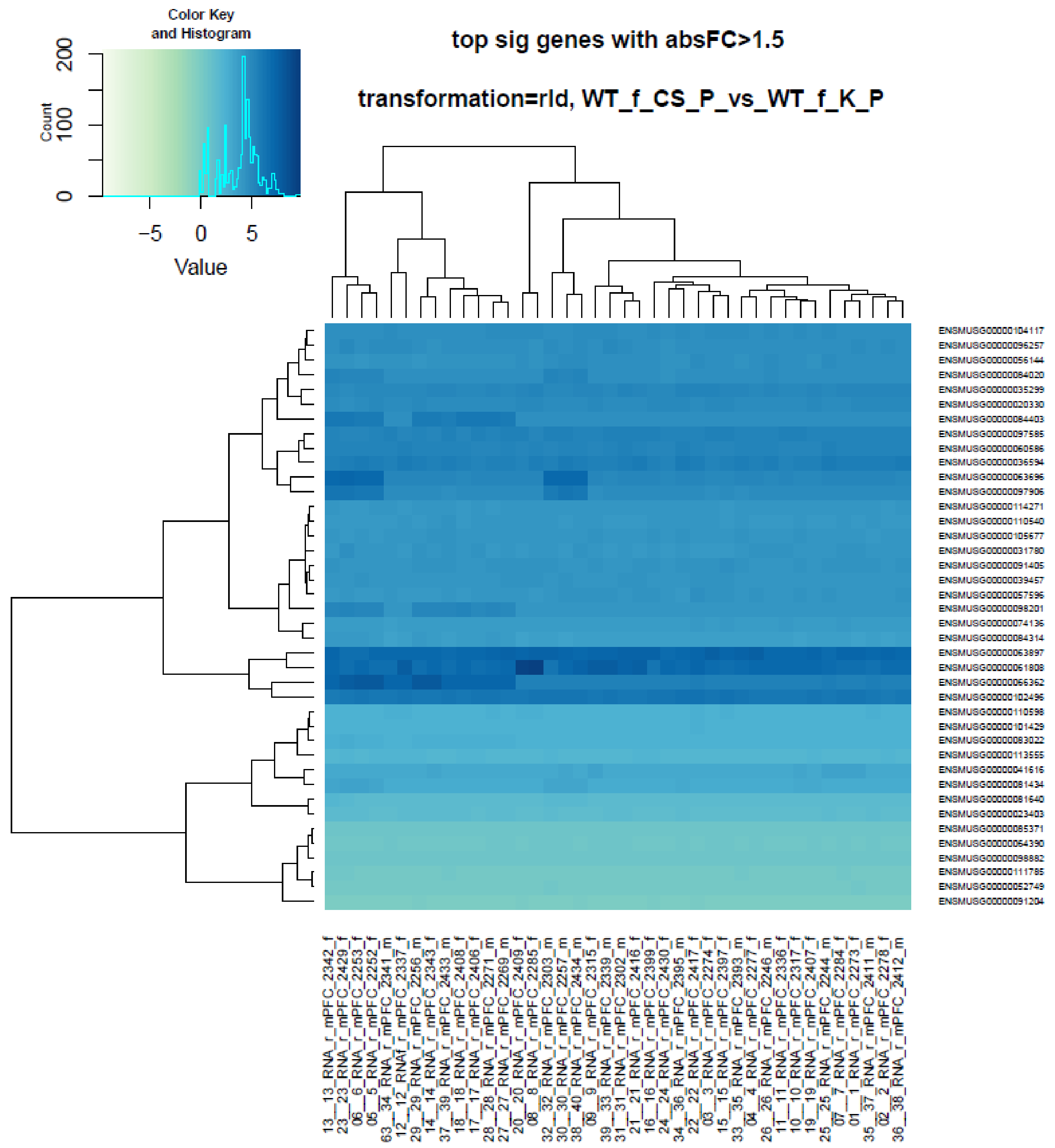

Suppl. Fig. 3 H

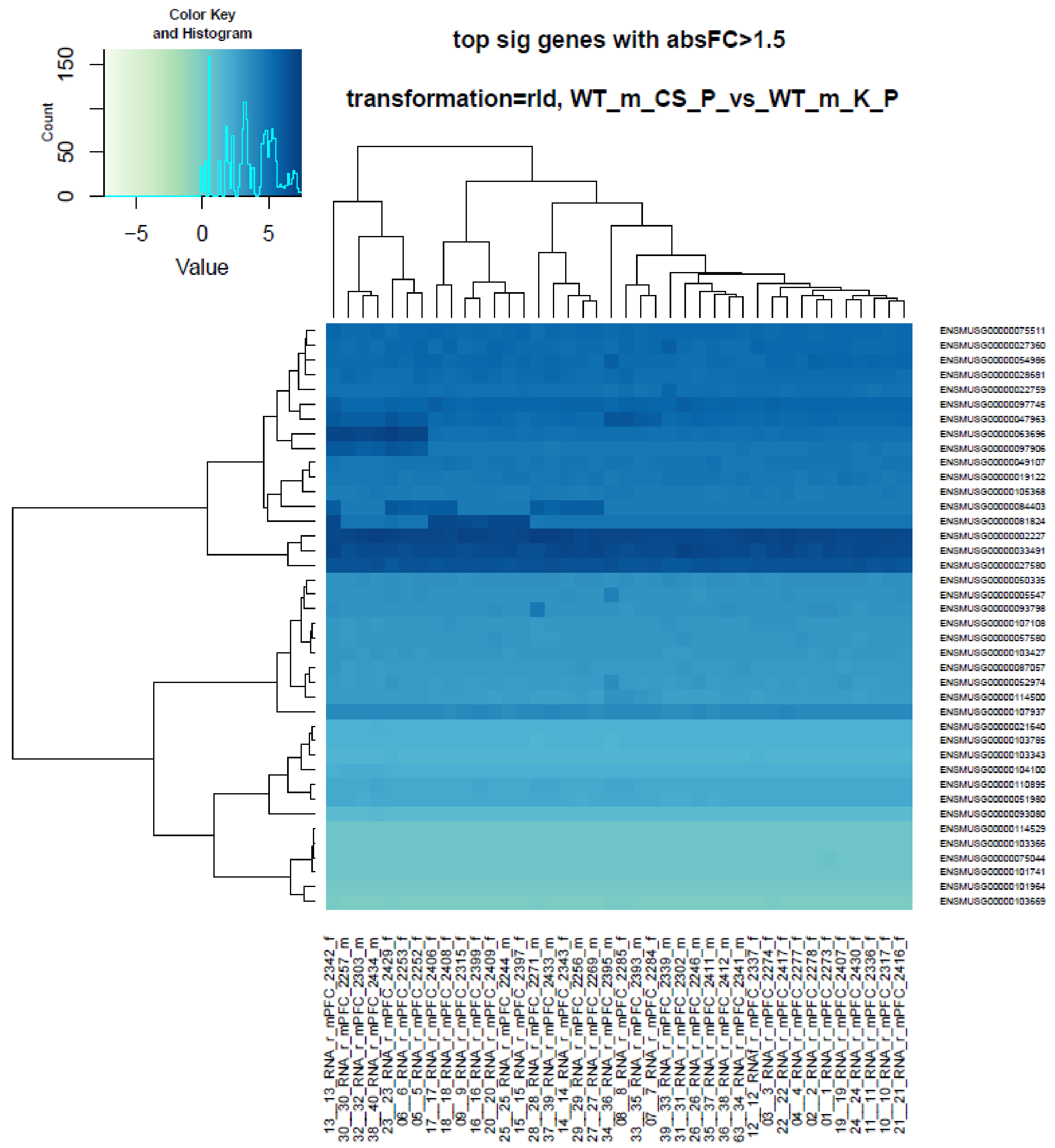

Supplemental Fig. 3 Heatmap and dendrogram illustrating prefrontal cortex genes in different group comparisons. Top significant genes (ensemble IDs; statistics: Wald test) with absolute Fold changes (FC) > 1.5 were plotted against RNA-seq based gene expression data (normalized (rld transformation) counts) from *Zdhhc7* mice (N=40) from eight groups (1. C-WT-m, N=4; 2. CS-WT-m, N=4; 3. C-KO-m, N=4; 4. CS-KO-m, N=4; 5. C-WT-f, N=6; 6. CS-WT-f, N=6; 7. C-KO-f, N=6; 8. CS-KO-f N=6); for individual sample allocation to groups see GEO data deposition (GSE281404). Individual group comparisons were displayed in different parts of **Fig.: A)** KO_f_CS_P_vs._KO_f_K_P, **B)** KO_f_CS_P_vs._WT_f_CS_P, **C)** KO_f_K_P_vs._WT_f_K_P, **D)** KO_m_CS_P_vs._KO_m_K_P, **E)** KO_m_CS_P_vs._WT_m_CS_P, **F)** KO_m_K_P_vs._WT_m_K_P, **G)** WT_f_CS_P_vs._WT_f_K_P, **H)** WT_m_CS_P_vs._WT_m_K_P.

Suppl. Fig. 4 A

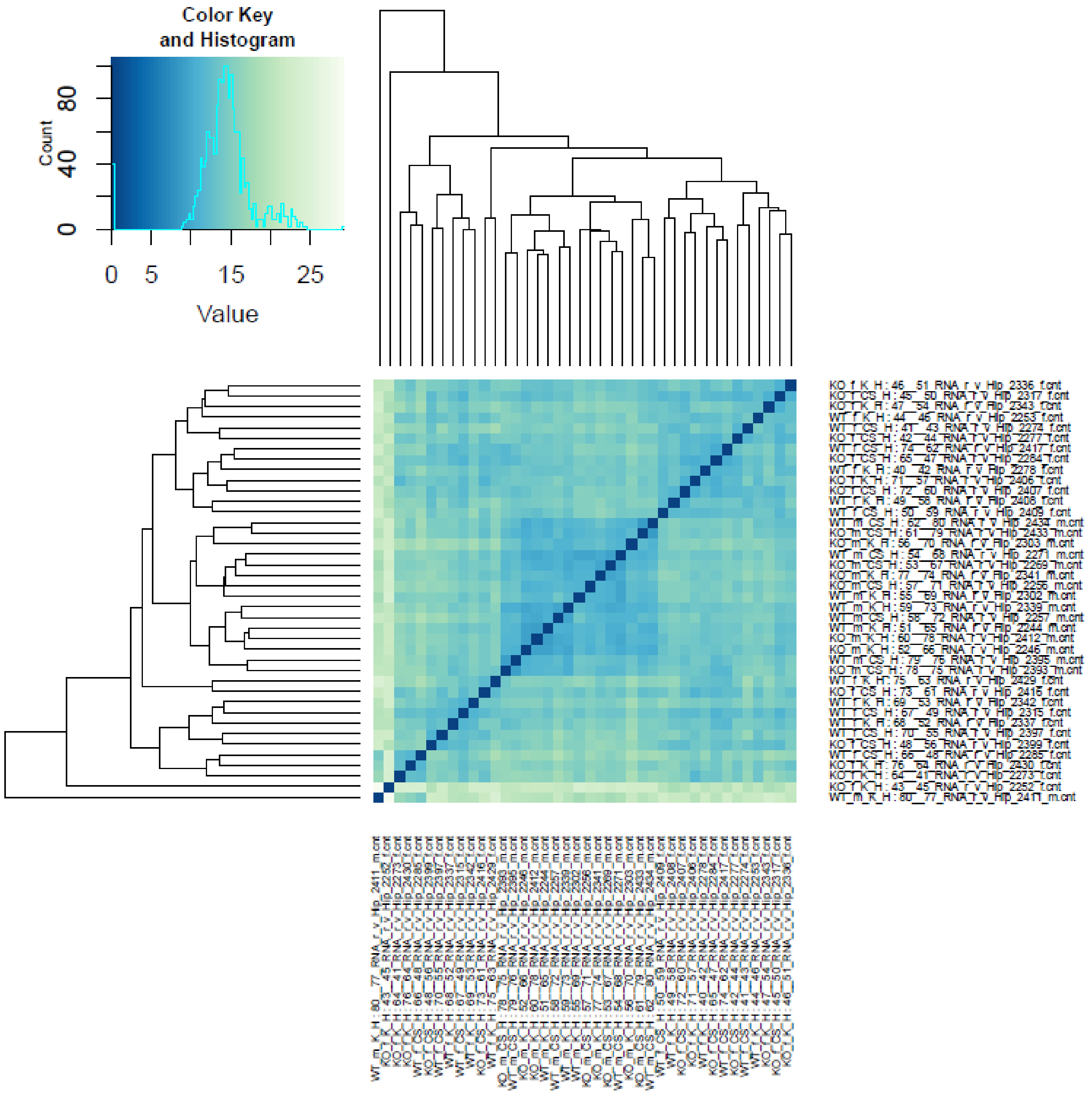

Suppl. Fig. 4 B

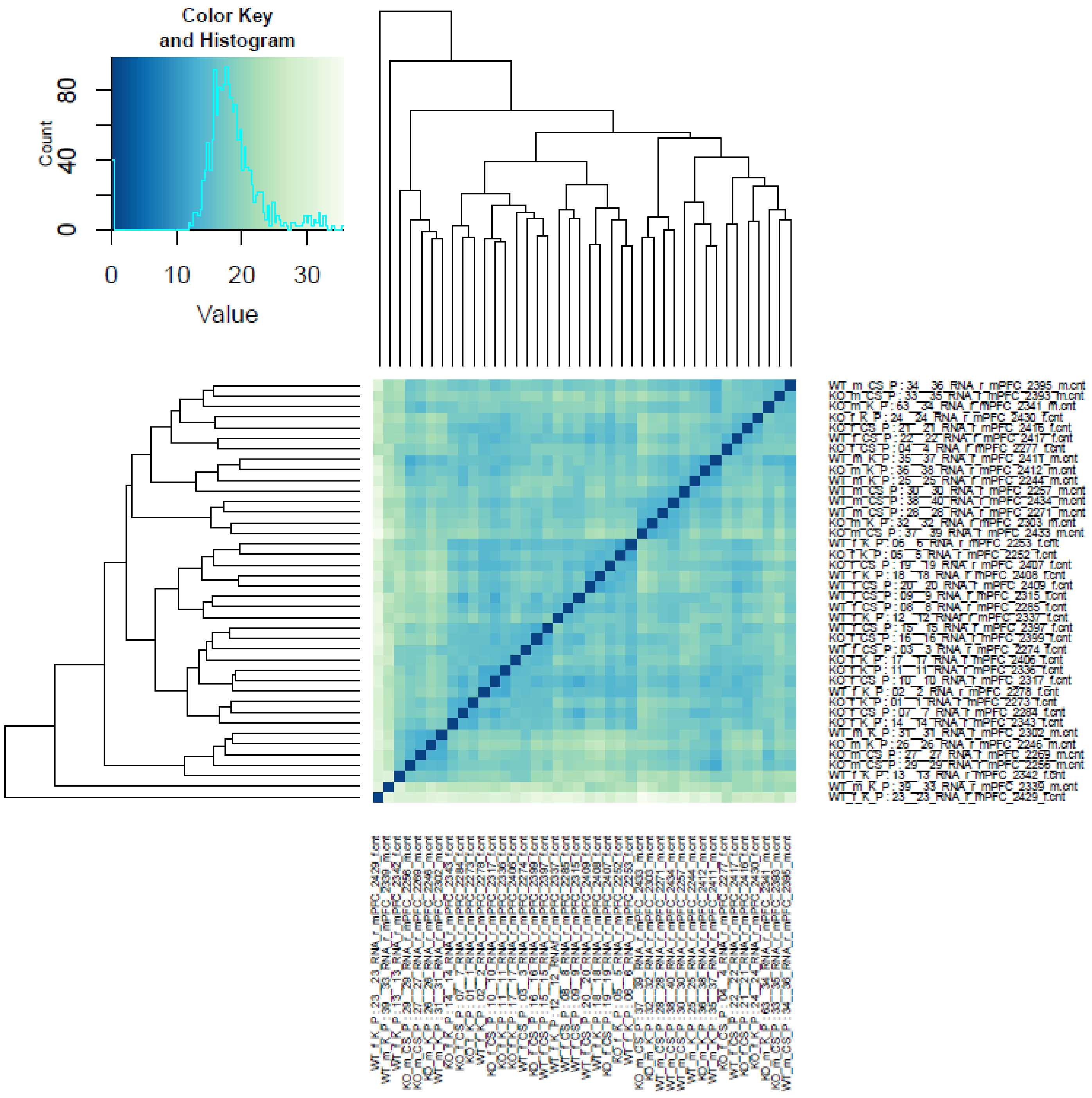

Supplemental Fig. 4 Heatmap and dendrogram illustrating hippocampal (Fig. A) and prefrontal cortex (Fig. B) gene counts sample by sample. RNA-seq based gene expression data (normalized (rld transformation) counts) from *Zdhhc7* mice (N=40) from eight groups (1. C-WT-m, N=4; 2. CS-WT-m, N=4; 3. C-KO-m, N=4; 4. CS-KO-m, N=4; 5. C-WT-f, N=6; 6. CS-WT-f, N=6; 7. C-KO-f, N=6; 8. CS-KO-f N=6) were plotted against each other; for individual sample allocation to groups see GEO data deposition (GSE281404).

Suppl. Fig. 5 A

volcano plot KO_f_CS_H_vs_KO_f_K_H

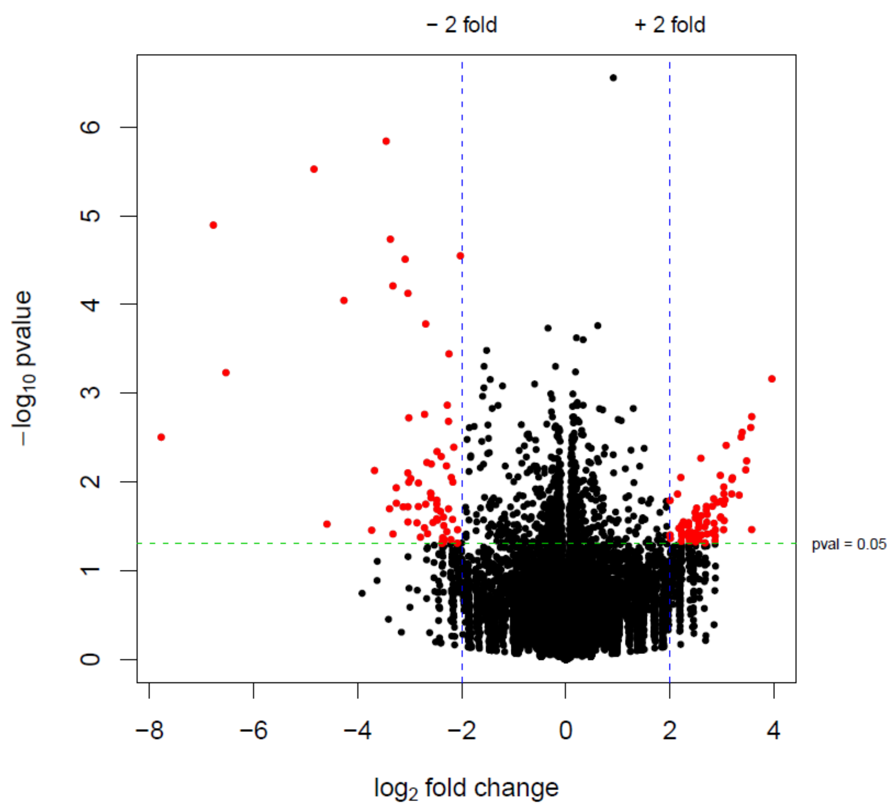

Suppl. Fig. 5 B

volcano plot KO_f_CS_H_vs_WT_f_CS_H

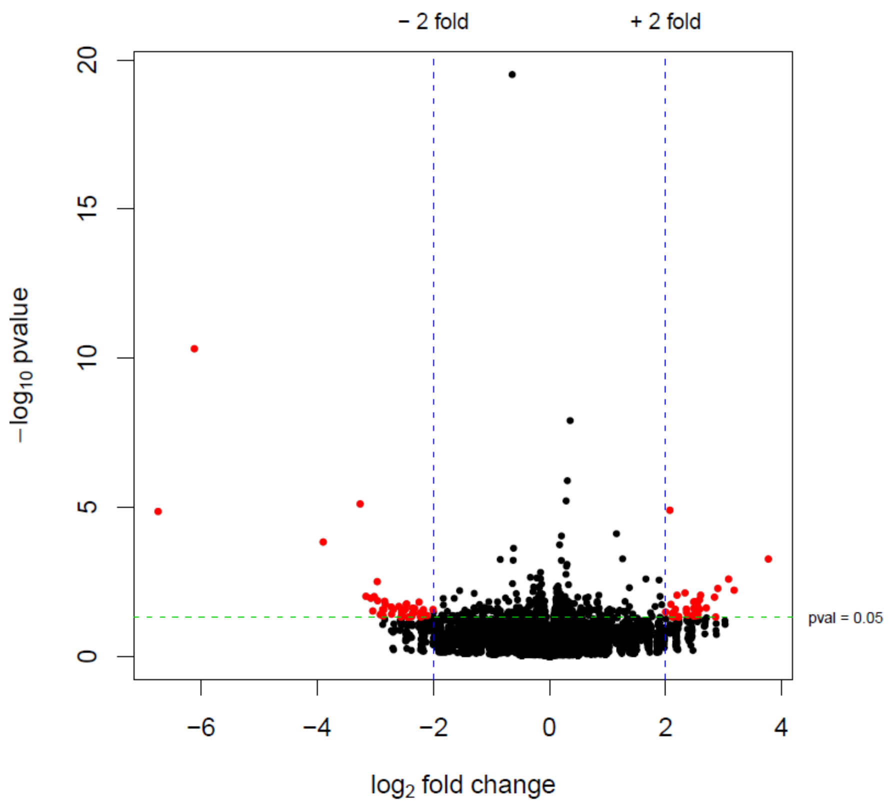

Suppl. Fig. 5 C

volcano plot KO_f_K_H_vs_WT_f_K_H

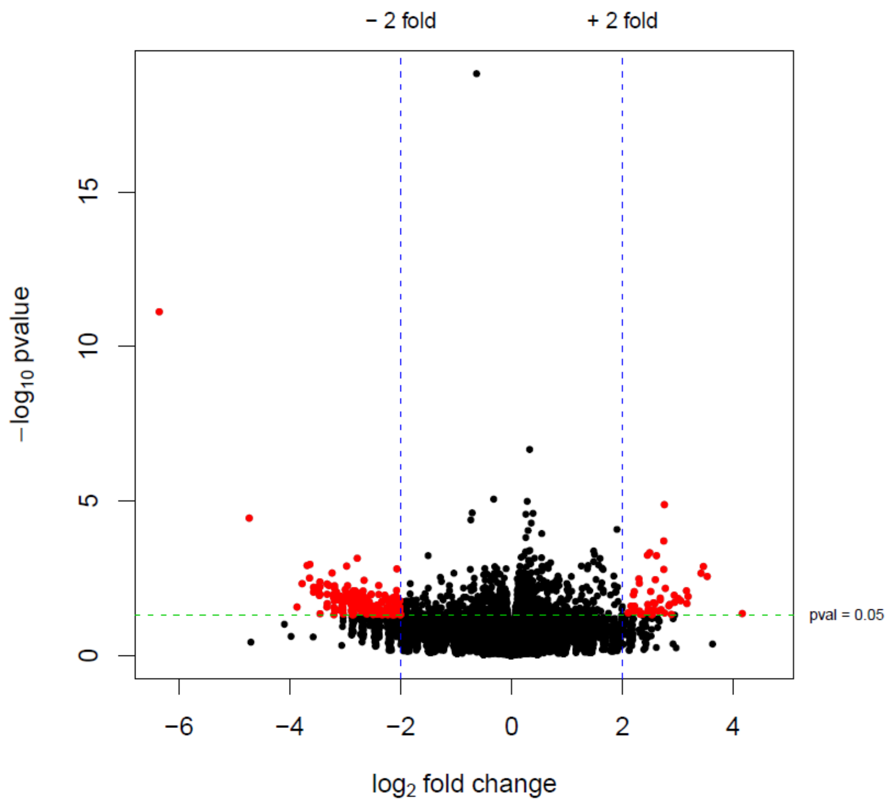

Suppl. Fig. 5 D

volcano plot KO_m_CS_H_vs_WT_m_CS_H

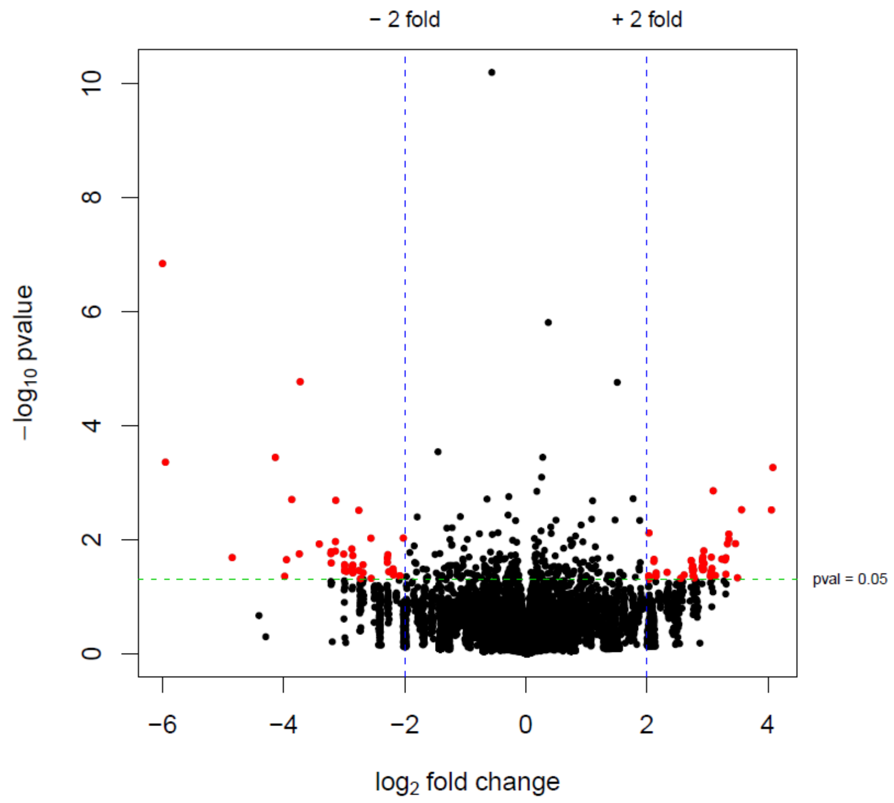

Supplemental Fig. 5 Volcanoplots illustrating hippocampal genes in different group comparisons: to be continued below

Suppl. Fig. 5 E

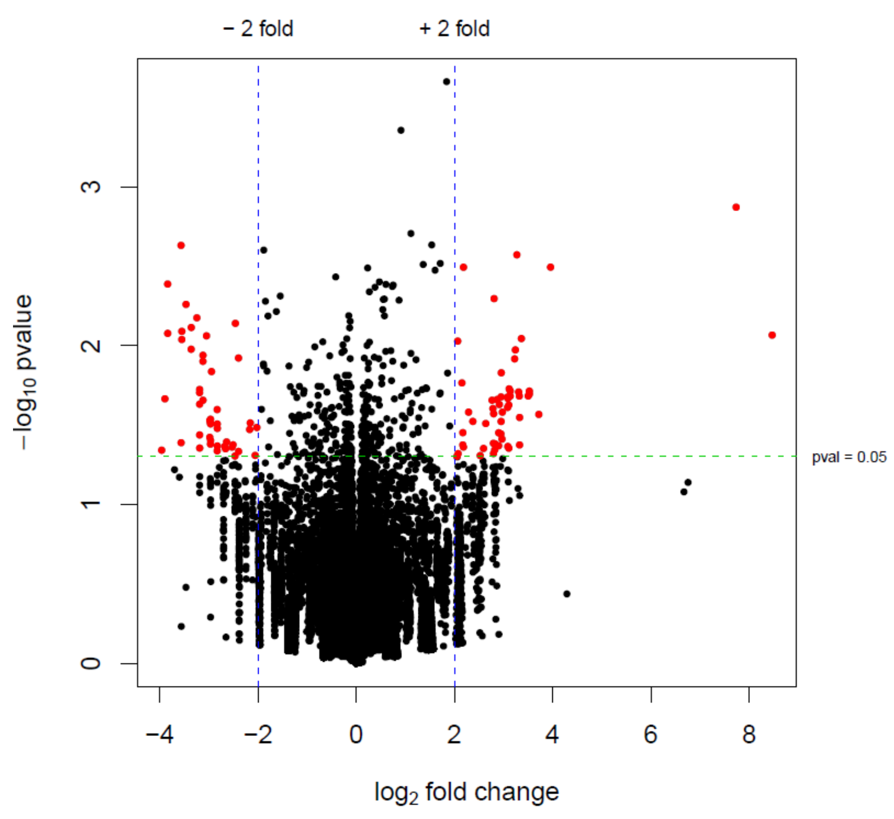

Suppl. Fig. 5 F

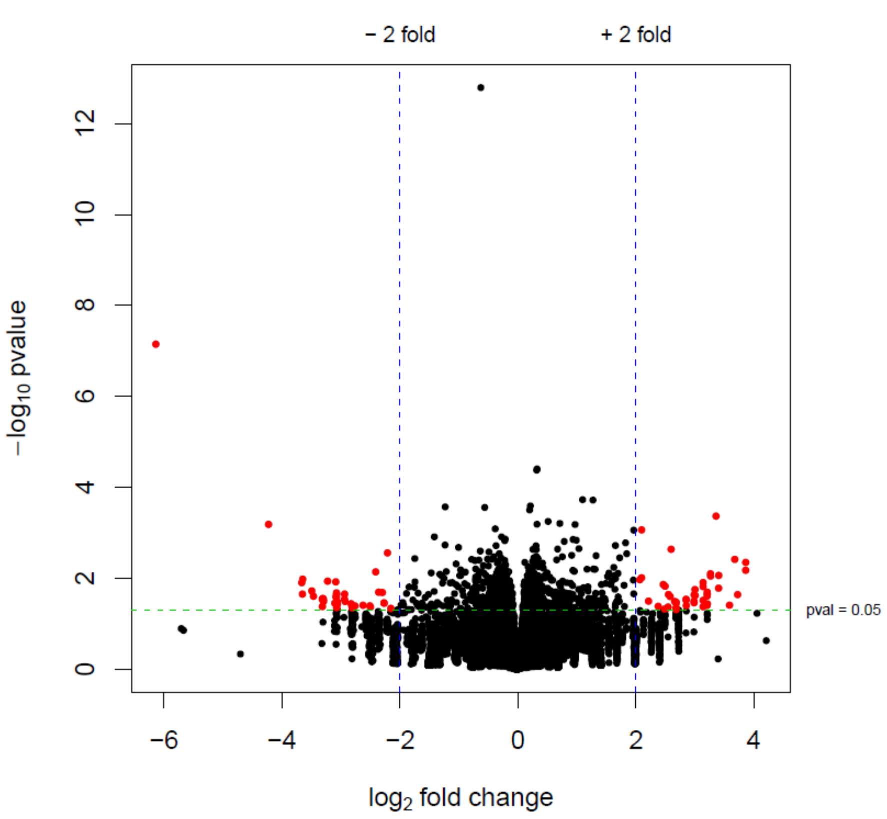

Suppl. Fig. 5 G

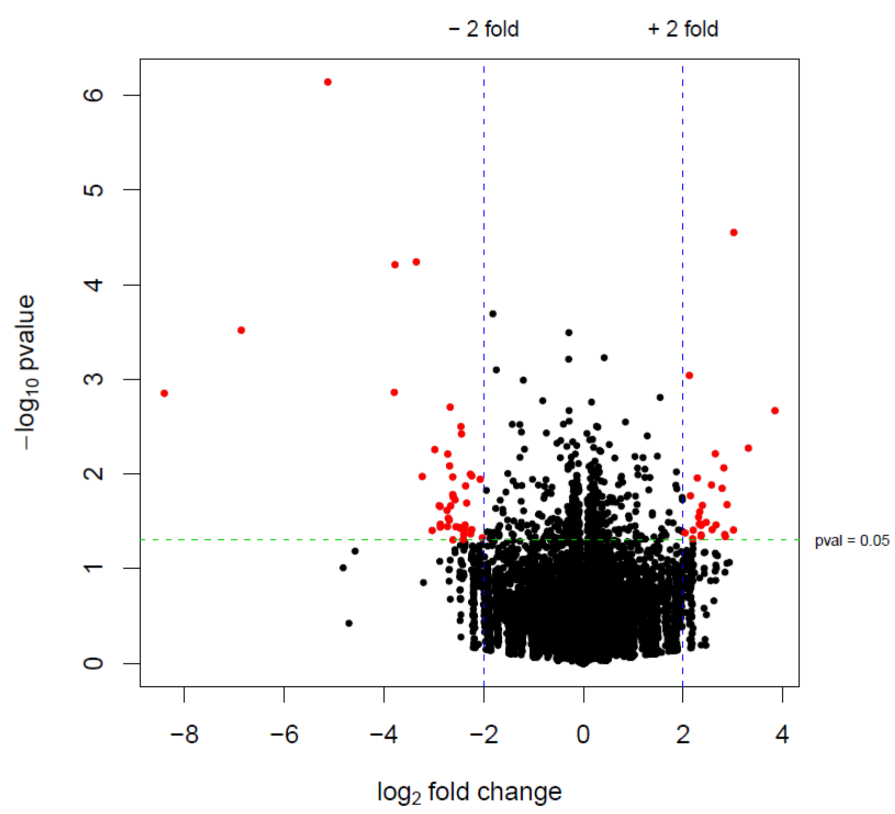

Suppl. Fig. 5 H

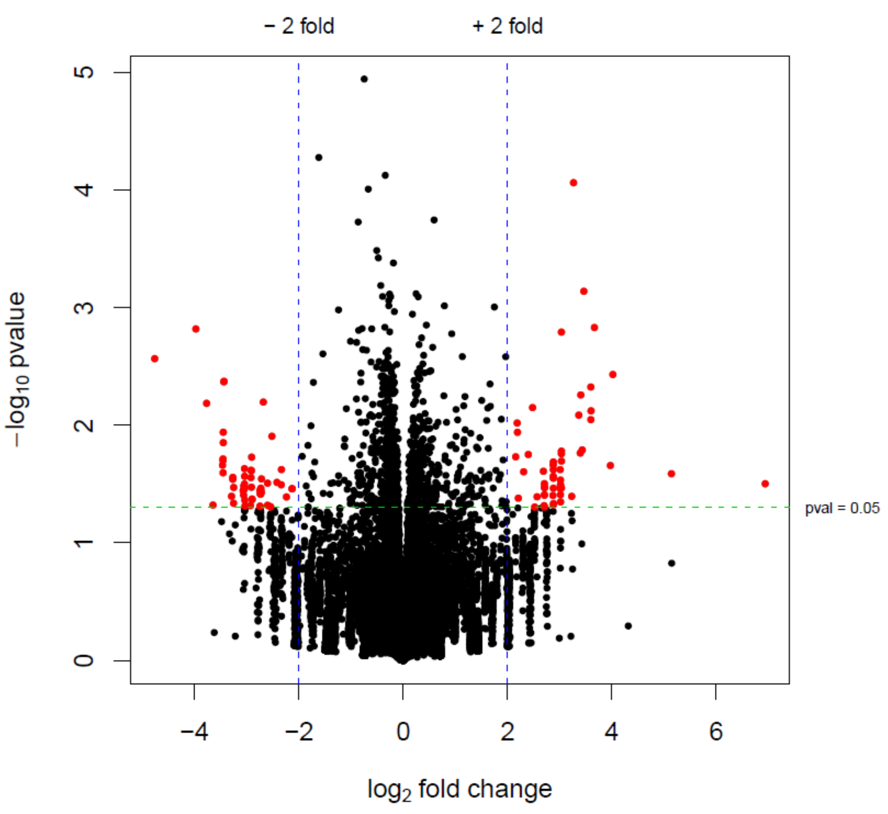

Supplemental Fig. 5 Volcanoplots illustrating hippocampal genes in different group comparisons. Differentially expressed genes via DESeq2 with top significant candidates highlighted in red (statistics: Wald test, alpha-level of significance (uncorrected for multiple testing): 0.05) are plotted against $\log_2$ fold change. Data were based on RNA-seq gene expression data (normalized (rld transformation) counts) from *Zdhhc7* mice (N=40) from eight groups (1. C-WT-m, N=4; 2. CS-WT-m, N=4; 3. C-KO-m, N=4; 4. CS-KO-m, N=4; 5. C-WT-f, N=6; 6. CS-WT-f, N=6; 7. C-KO-f, N=6; 8. CS-KO-f N=6); for individual sample allocation to groups see GEO data deposition (GSE281404). Individual group comparisons were displayed in different parts of **Fig.: A)** KO_f_CS_H_vs._KO_f_K_H, **B)** KO_f_CS_H_vs._WT_f_CS_H, **C)** KO_f_K_H_vs._WT_f_K_H, **D)** KO_m_CS_H_vs._WT_m_CS_H, **E)** KO_m_CS_H_vs._KO_m_K_H, **F)** KO_m_K_H_vs._WT_m_K_H, **G)** WT_f_CS_H_vs._WT_f_K_H, **H)** WT_m_CS_H_vs._WT_m_K_H.

Suppl. Fig. 6 A

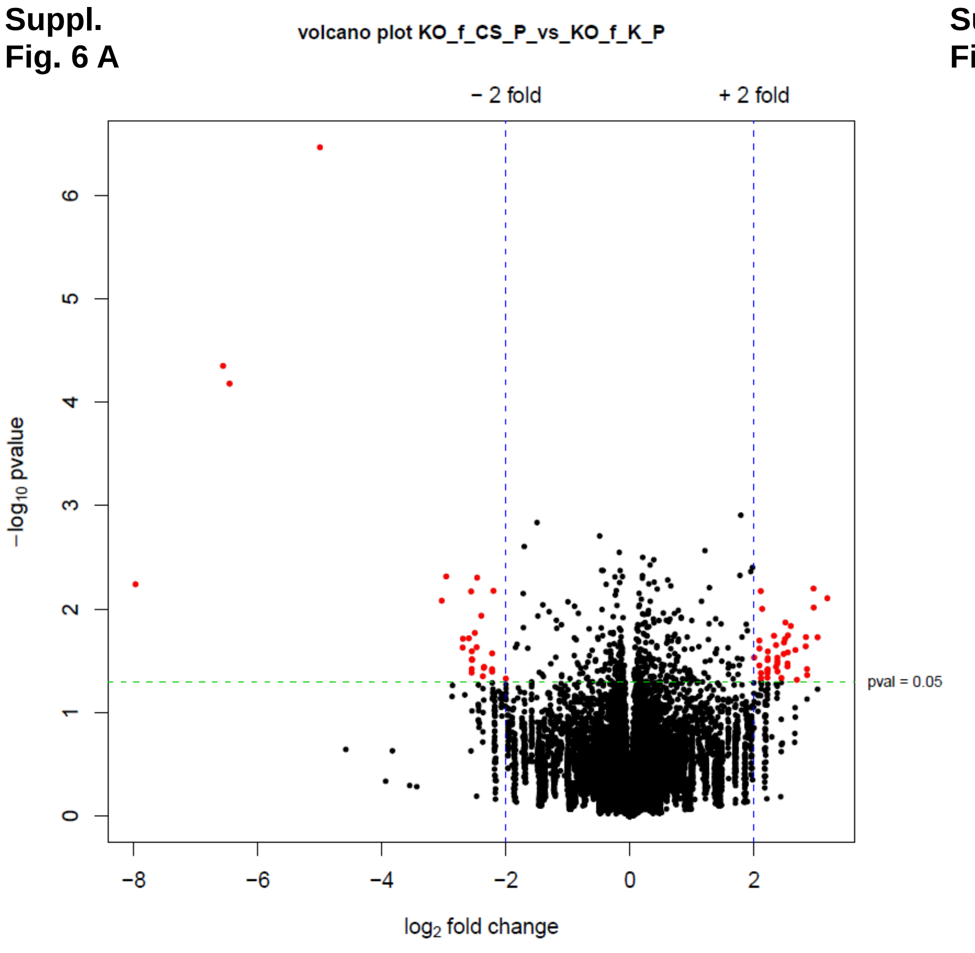

Suppl. Fig. 6 B

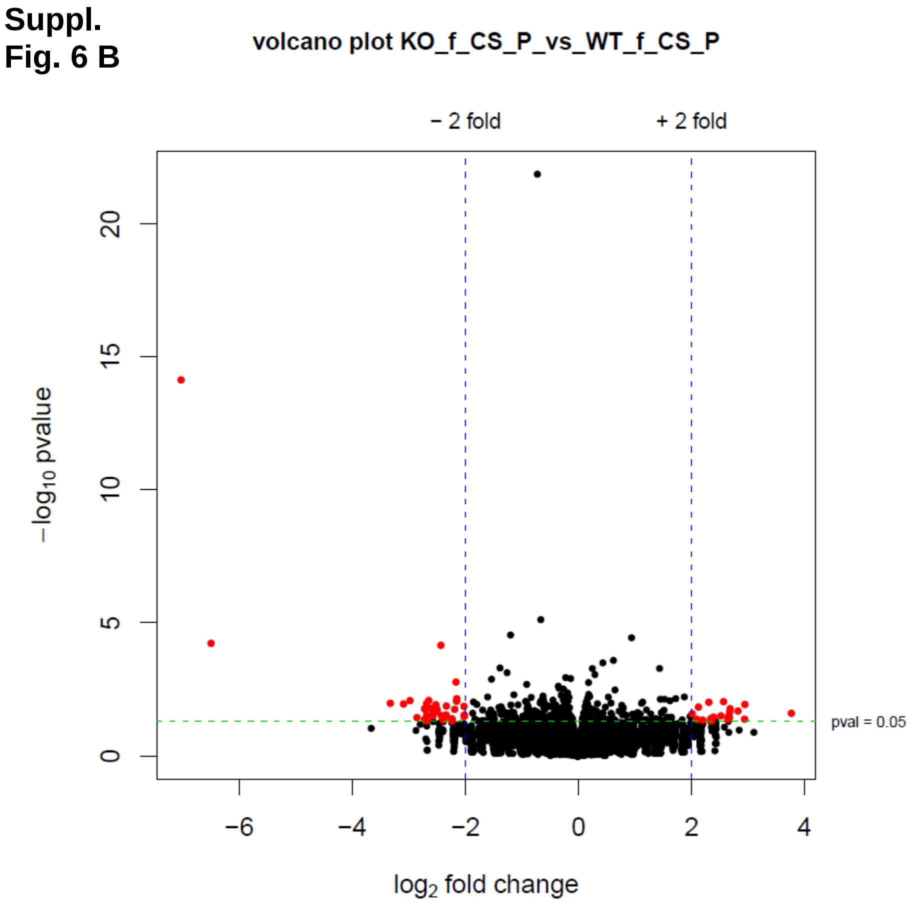

Suppl. Fig. 6 C

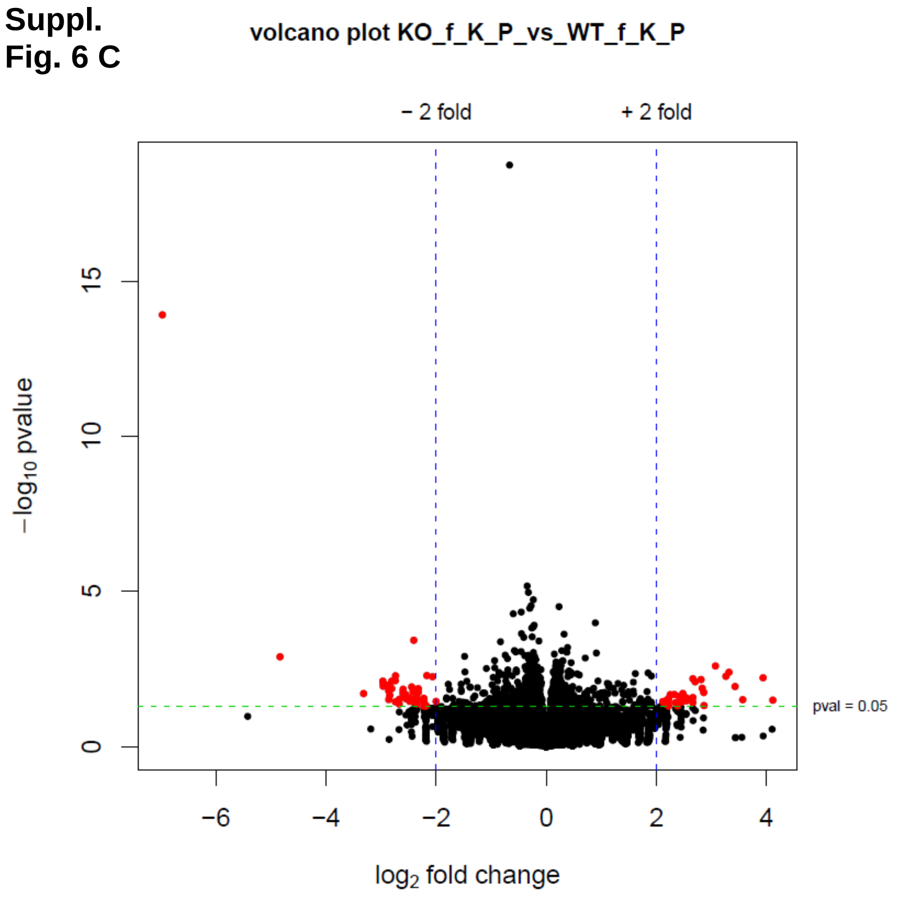

Suppl. Fig. 6 D

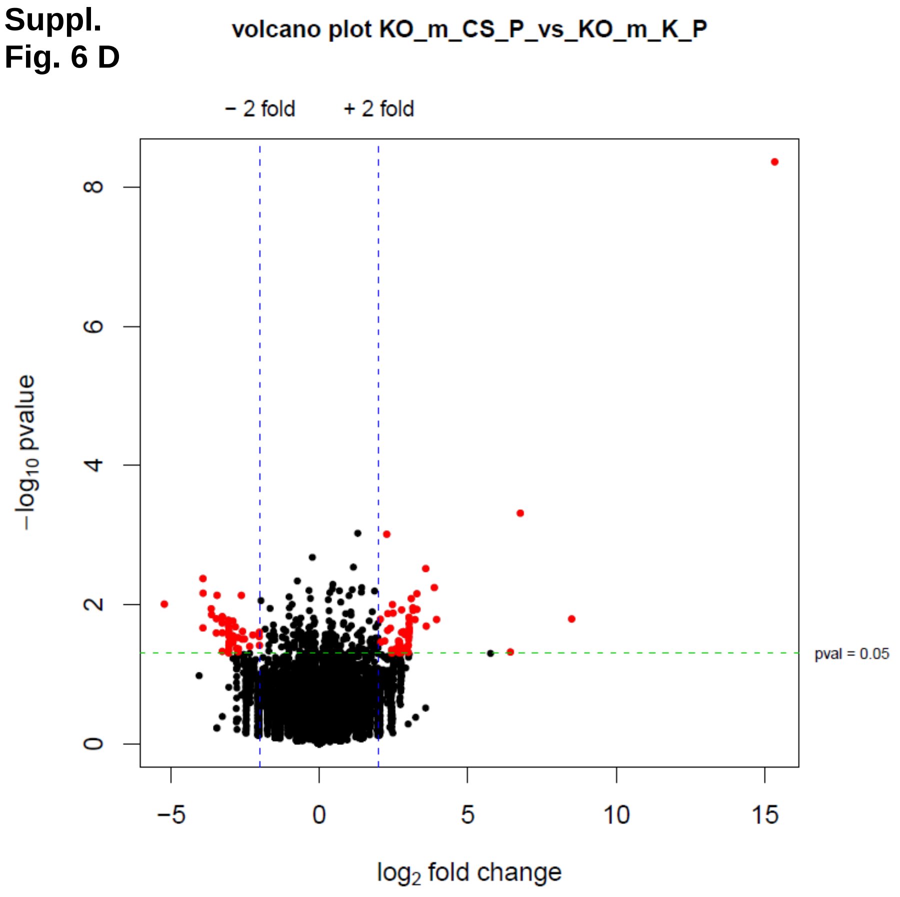

Supplemental Fig. 6 Volcanoplots illustrating prefrontal cortex genes in different group comparisons: to be continued below

Suppl. Fig. 6 E

volcano plot KO_m_CS_P_vs_WT_m_CS_P

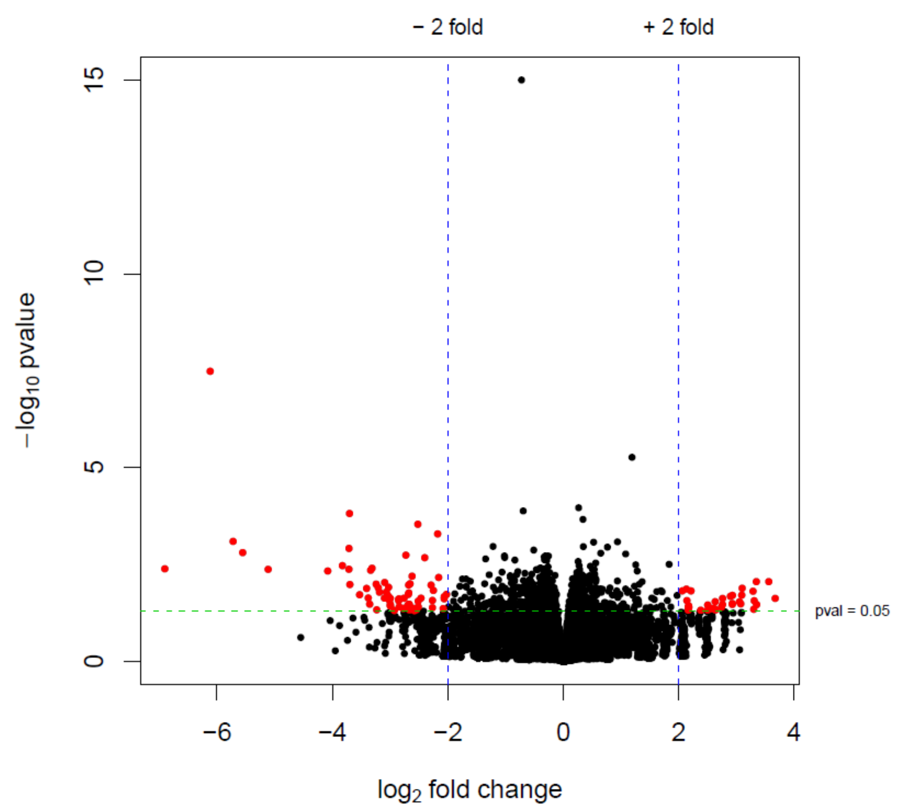

Suppl. Fig. 6 F

volcano plot KO_m_K_P_vs_WT_m_K_P

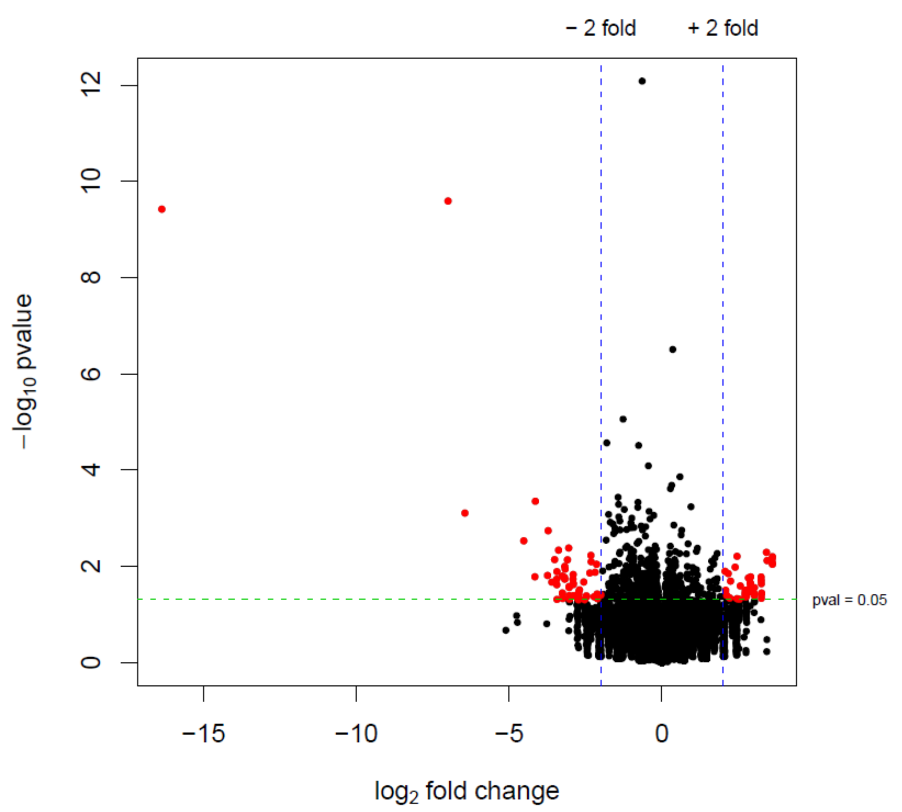

Suppl. Fig. 6 G

volcano plot WT_f_CS_P_vs_WT_f_K_P

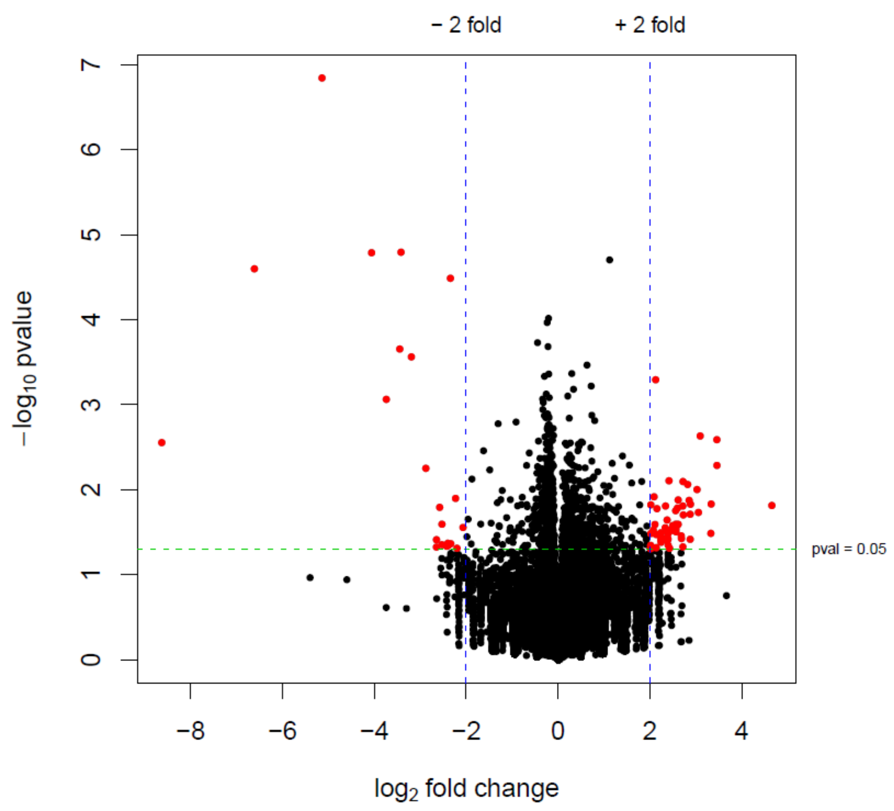

Suppl. Fig. 6 H

volcano plot WT_m_CS_P_vs_WT_m_K_P

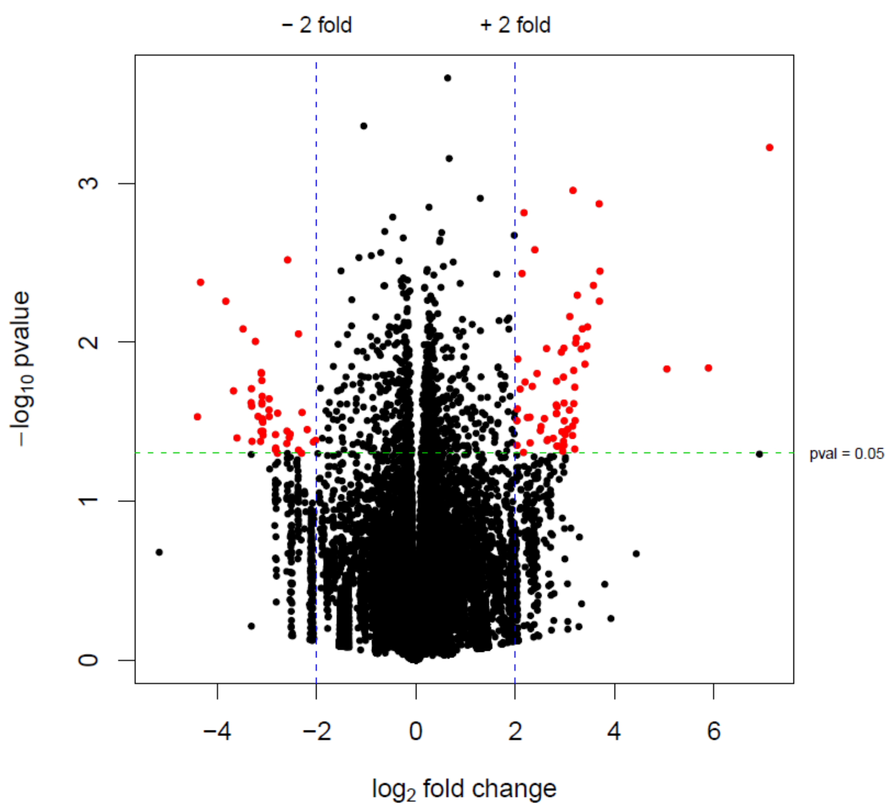

Supplemental Fig. 6 Volcanoplots illustrating prefrontal cortex genes in different group comparisons. Differentially expressed genes via DESeq2 with top significant candidates highlighted in red (statistics: Wald test, alpha-level of significance (uncorrected for multiple testing): 0.05) are plotted against log_2 fold change. Data were based on RNA-seq gene expression data (normalized (rld transformation) counts) from *Zdhhc7* mice (N=40) from eight groups (1. C-WT-m, N=4; 2. CS-WT-m, N=4; 3. C-KO-m, N=4; 4. CS-KO-m, N=4; 5. C-WT-f, N=6; 6. CS-WT-f, N=6; 7. C-KO-f, N=6; 8. CS-KO-f N=6); for individual sample allocation to groups see GEO data deposition (GSE281404). Individual group comparisons were displayed in different parts of **Fig.: A)** KO_f_CS_P_vs._KO_f_K_P, **B)** KO_f_CS_P_vs._WT_f_CS_P, **C)** KO_f_K_P_vs._WT_f_K_P, **D)** KO_m_CS_P_vs._KO_m_K_P, **E)** KO_m_CS_P_vs._WT_m_CS_P, **F)** KO_m_K_P_vs._WT_m_K_P, **G)** WT_f_CS_P_vs._WT_f_K_P, **H)** WT_m_CS_P_vs._WT_m_K_P.
